# Causal relationship between immune cells and bladder cancer: A bidirectional Mendelian randomization study

**DOI:** 10.1097/MD.0000000000044268

**Published:** 2025-09-26

**Authors:** Anneng Hu, Chunyan Hu, Yuhang Lin, Xiaole Zhu, Junyang Li, Fuwen Luo, Xiaodong Yu

**Affiliations:** a Department of Urology, Affiliated Hospital of North Sichuan Medical College, Nanchong, Sichuan, PR China.

**Keywords:** bladder cancer, genome-wide association, immune, immune cells, Mendelian randomization

## Abstract

Background and Objectives: Prior research has examined immune cell involvement in bladder cancer (BCa). This Mendelian randomization (MR) study investigated causal links between 731 immune cell traits and BCa risk, identifying potential immunotherapy targets. Immune cell data came from a Sardinian cohort (3757 individuals), and BCa data from genome-wide association studies. We used inverse variance weighting as the main analysis, with MR-Egger and weighted median methods for validation. Stability checks included sensitivity analyses. Sixteen immune traits (related to 4 immune signals: MFI, RC, AC, MP) showed significant BCa associations. Five increased risk (OR > 1, *P* < .05), while 11 were protective (OR < 1, *P* < .05). No reverse causality, heterogeneity, or horizontal pleiotropy was found (*P* > .05). This MR analysis demonstrates complex causal relationships between immune cell traits and BCa, offering new insights for immunotherapy and prevention.

## 1. Introduction

Globally, bladder cancer (BCa) is a major public health problem due to its complicated epidemiology, which is influenced by a combination of lifestyle, environmental, and genetic variables. This disease ranks as the second most prevalent urological cancer worldwide, responsible for 5,49,000 new cases and around 2,00,000 deaths annually.^[1]^ BCa incidence varies geographically for a variety of reasons, such as differences in industrialization, pollution in the environment, smoking rates, food preferences, and access to treatment. Industrialized areas are more likely to be exposed to occupational carcinogens including industrial chemicals, polycyclic aromatic hydrocarbons, and aromatic amines.^[2]^ Developed countries tend to report greater incidence rates than emerging regions. There are 2 types of BCa: muscle invasive bladder cancer (MIBC) and non-MIBC. This classification is based on whether the bladder’s muscular layer has been affected by the tumor. Research has indicated that whereas the remaining instances of BCa are MIBC, over 75% are non-MIBC.^[3]^ Histological subtypes of BCa consist of urothelial carcinoma, squamous cell carcinoma and adenocarcinoma.^[4]^

There is growing evidence that immune cells had a role in the development of BCa.^[5–10]^ The immune system actively regulates tumor growth through both pro-tumorigenic and anti-tumorigenic mechanisms. CD4+ and CD8+ T lymphocytes, along with natural killer cells, contribute to antitumor immunity by recognizing and eliminating cancerous cells.^[11,12]^ Conversely, tumor-associated macrophages and other immunosuppressive immune cells may inhibit antitumor responses and promote tumor progression when overactivated.^[13]^ The particular roles of different immune cell subtypes and whether there is a causal connection between these cells and tumor growth are still unknown, despite a basic understanding of the roles played by some immune cell types in the pathogenesis of prostate cancer.

While observational studies have suggested associations between immune cell phenotypes and BCa, these findings are susceptible to confounding and reverse causality, limiting their ability to establish causation. To address this limitation, Mendelian randomization (MR) offers a powerful approach by leveraging genetic variants as instrumental variables (IVs) to infer causal relationships.^[14–16]^ MR mimics a randomized controlled trial by minimizing confounding effects and eliminating reverse causation bias. Study employs a bidirectional MR framework to investigate whether specific immune cell phenotypes have a causal impact on BCa risk. By identifying causal relationships, our findings could provide novel insights into immunotherapeutic targets and strategies for BCa prevention and treatment.

## 2. Materials and methods

### 2.1. Data sources

A population-based immune profile analysis published in the journal Nature Genetics was used in this investigation. 3757 Sardinian citizens comprised the group used in the study. A broad spectrum of 731 immunophenotypes (The genome-wide association studies [GWAS] catalog [GCST90001391 to GCST90002121]; 7 groups) were covered by the comprehensive investigation, involving relative cell counts (n = 92), morphological characteristics (n = 32), median fluorescence intensities (n = 389), and absolute cell counts (n = 118).^[17]^

**Figure 1.**
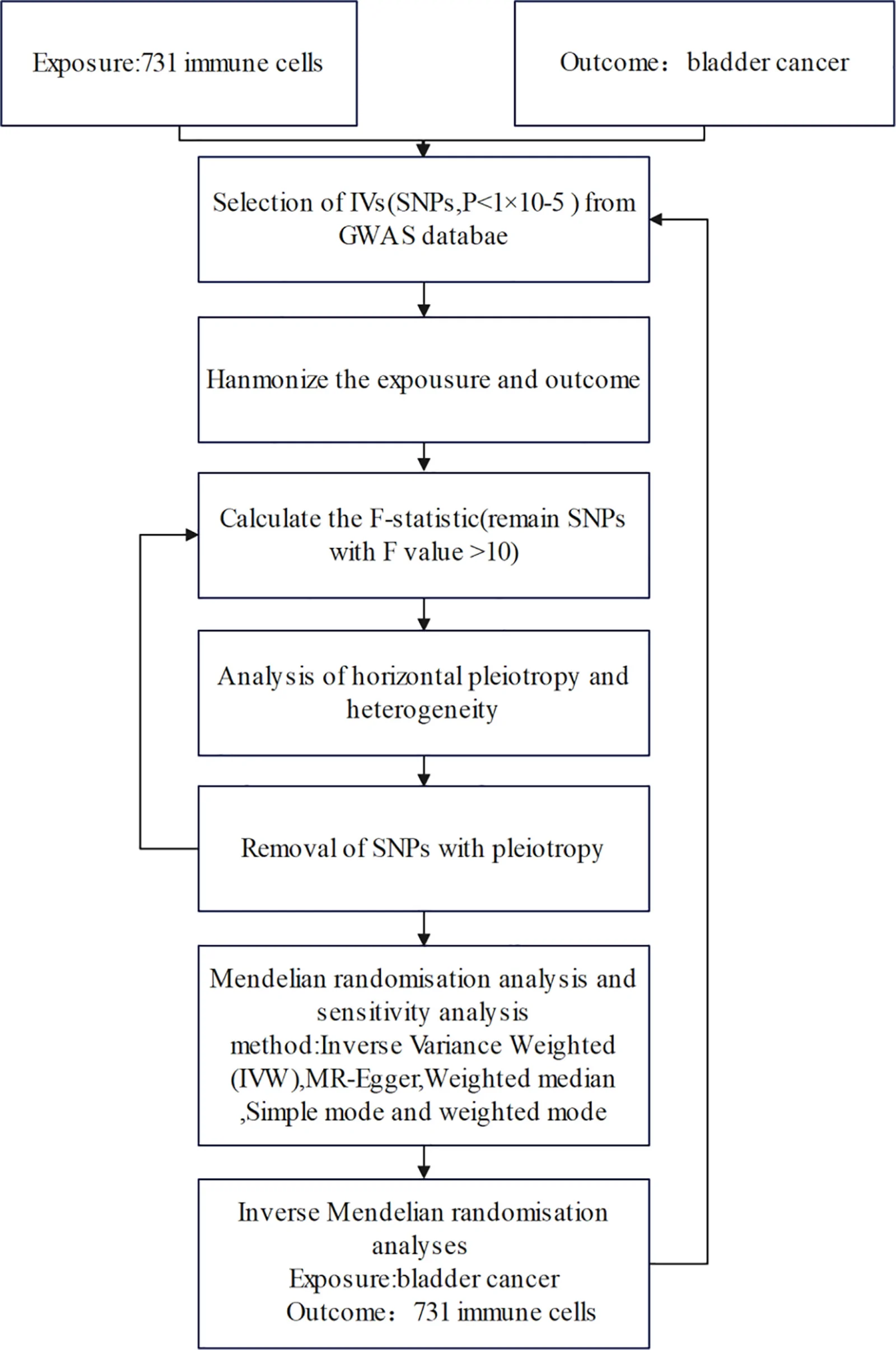
Flowchart of a MR study between 731 immune cells and bladder cancer. GWAS = genome-wide association studies, IVs = instrumental variables, IVW = inverse variance weighted, MR = Mendelian randomization, SNPs = single-nucleotide polymorphisms.

**Figure 2.**
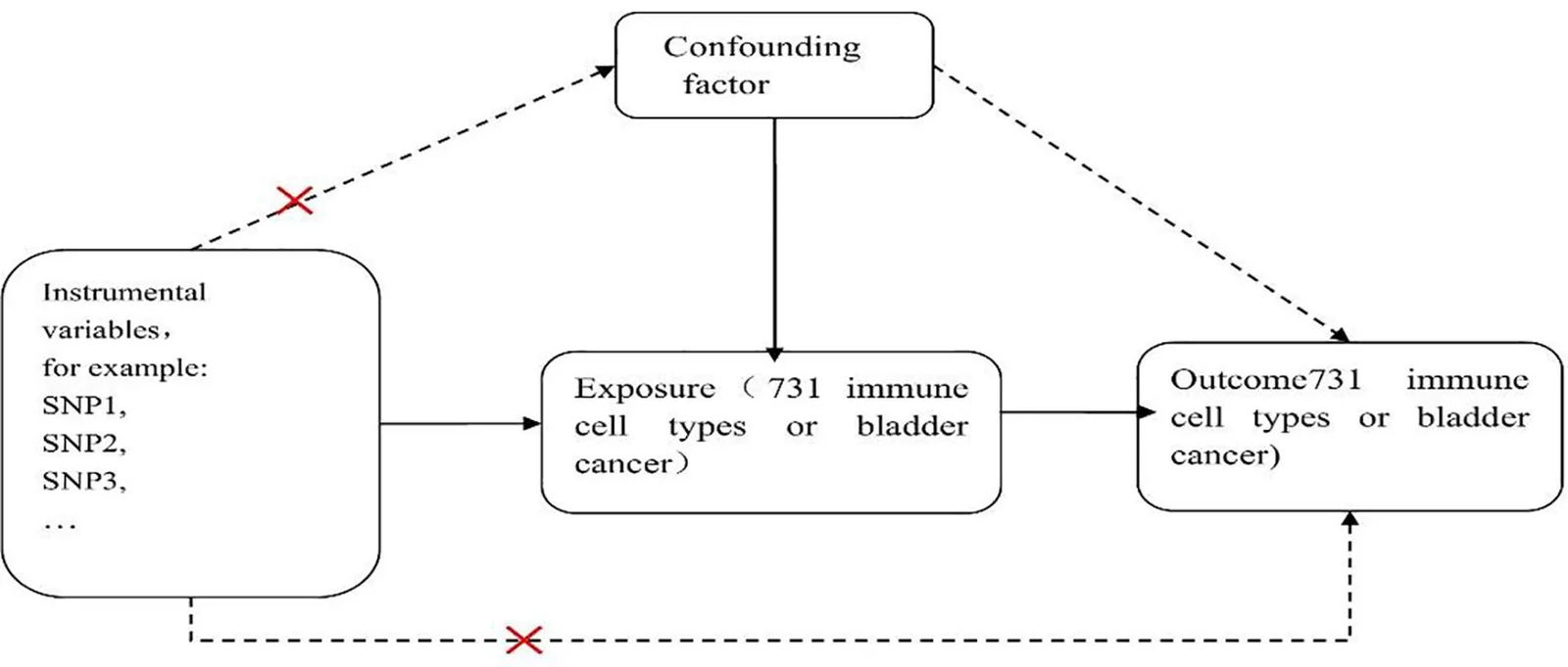
The 3 major assumptions of Mendelian randomization. (1) IVs are linked to risk exposure. (2) There is no connection between IVs and any confounding variables that affect the exposure-outcome link. (3) IVs cannot influence the result in any other manner than through the exposure. IVs = instrumental variables, SNP = single-nucleotide polymorphism.

The BCa data used in this study were sourced from the Integrative Epidemiology Unit Open GWAS database (https://gwas.mrcieu.ac.uk/). The study included 1279 European BCa patients as the study group and 3,72,016 European people without BCa as the control group. A total of 99,04,926 SNPs were screened for their impact on prostate cancer. In order to rigorously assess the potential causal relationship between the 731 immune cells and BCa, we designed a flowchart for a MR study that met these assumptions (Fig. 1). Figure 2 illustrates the study’s specific research approach, and Table 1 provides specific details on data sources and features. The established standards in endoscopy, imaging, and histology pathology determine the diagnostic criteria for BCa.

**Table 1:** Comprehensive details on the data analysis.

Exposure or outcome	Sample size	Population	Data source	PMID
Immune cell types	3757	European	https://pubmed.ncbi.nlm.nih.gov/32929287/	32929287
BCa	1279	European	https://gwas.mrcieu.ac.uk/datasets/ieu-b-4874/	–

BCa = bladder cancer.

### 2.2. Study design

In order to figure out which immune cell morphologies may be causally related to the risk of BCa, we first use these phenotypes as the exposure. We then evaluate the possible reverse causal links with immune cell morphologies using BCa as the exposure. In the study, single nucleotide polymorphisms, or SNPs, are used as IVs. Three essential presumptions are met by the chosen IVs: Relevance assumption: IVs are linked to risk exposure. Independence assumption:There is no connection between IVs and any confounding variables that affect the exposure-outcome link. Exclusion restriction assumption: IVs cannot influence the result in any other manner than through the exposure.^[18]^ IVs that don’t adhere to the 3 main presumptions won’t be allowed.

### 2.3. Selection of IVs

First, the criteria of *P* value < 1 × 10^−5^ was fulfilled by filtering the GWAS data to include associated SNPs.^[19]^ In addition, the parameter *r* threshold was set to 0.001 and the SNPs’ distance was adjusted to 10,000 kb for the study in order to prevent linkage disequilibrium of SNPs from influencing the results. Second, the PhenoScanner V2 database was utilized to confirm if any other confounding factors were connected to the previously indicated included SNP sites. Lastly, the *F* statistic (defined as *F* = β^2^/SE^2^, where β is the allelic effect value and SE is the standard error) was used to eliminate *F* values with a value <10 in order to evaluate whether the included SNPs were affected by weak IVs. The SNPs were eliminated to prevent any influence on the results if their *F* statistic was <10, which suggested that they may have weak IV bias. Following that, the IEU OpenGWAS database or the FinnGen database was used to extract the outcome information, from which the correlations between SNPs that supported the hypothesis were derived. The datasets that were exposed and the outcome were combined, and the palindromic sequences were eliminated. Those last IVs for the exposure were the SNPs that remained.

### 2.4. Statistical analysis

“TwoSampleMR (v.0.6.2),” “ieugwasr (1.0.0),” “ggplot2 (3.5.1.9),” and “MR-PRESSO (1.0)” packages in R (v.4.3.2) were used to carry out the MR analysis in this work.MR first used the TwoSampleMR software to analyze the screened IVs after they were retrieved from the ending factors. There were 5 popular approaches for MR analysis that were used: MR-Egger regression test – used to assess and correct for horizontal pleiotropy. Inverse variance weighted (IVW) method – assumes all IVs are valid instruments and provides the most efficient estimate. Weighted median method – provides a robust causal estimate even when up to 50% of IVs are invalid. Simple mode and weighted mode methods – used as supplementary approaches for additional validation. IVW was the primary analytical method and was supported by other ones. Because it employs the quadratic of se, which is the inverse of the ending variance, as the weight for the fit, the IVW methodology is distinguished by its refusal to consider the existence of an intercept term.^[20]^ A number of sensitivity studies were carried out to better account for potential pleiotropy. The findings of the MR analysis were then exposed to sensitivity analyses, including the horizontal multiple validity examination and the heterogeneity test. Weighted linear regression with intercepts, as proposed by MR-Egger,^[21]^ was utilized to evaluate the presence of horizontal multiplication among the IVs, and Cochran’s *Q* test^[22]^ was performed to quantify the heterogeneity of the IVs, with *P* < .05 showing the presence of heterogeneity. Furthermore, the leave-one-out sensitivity test was employed to evaluate the potential substantial effects of a single SNP on the causative effect. The study’s dependability and rigor are further improved by the application of many statistical approaches, which also contribute to clarifying the complex interaction between immune cells and BCa. *P* < .05 have been considered statistically significant. All data are displayed as odds ratios (OR) and 95% confidence intervals.

The STROBE-MR statement^[23]^ and MR investigations Guidelines^[24]^ were followed in the conduct of this MR study. Supplementary File 1, Supplemental Digital Content, https://links.lww.com/MD/Q192 lists STROBE-MR checklist. This study exclusively used publicly available GWAS data and did not involve individual participants, thus requiring no additional ethical approval. However, we ensured full compliance with the ethical guidelines set by the GWAS data providers. Additionally, our study adhered to the Declaration of Helsinki and relevant privacy protection regulations to safeguard participant rights.^[25]^

## 3. Results

### 3.1. Immunophenotypes’ causative relationship to bladder cancer

The primary findings of the investigation into the relationship between the risk of BCa and 731 immune cell types. Sixteen immune cell traits involving the 4 immunological signal types (MFI, RC, AC, and MP) were shown to be significantly correlated with the risk of BCa in our investigation. Tables S1 and S2, Supplemental Digital Content, https://links.lww.com/MD/Q194 list the IVs used for immunological characteristics.

**Figure 3.**
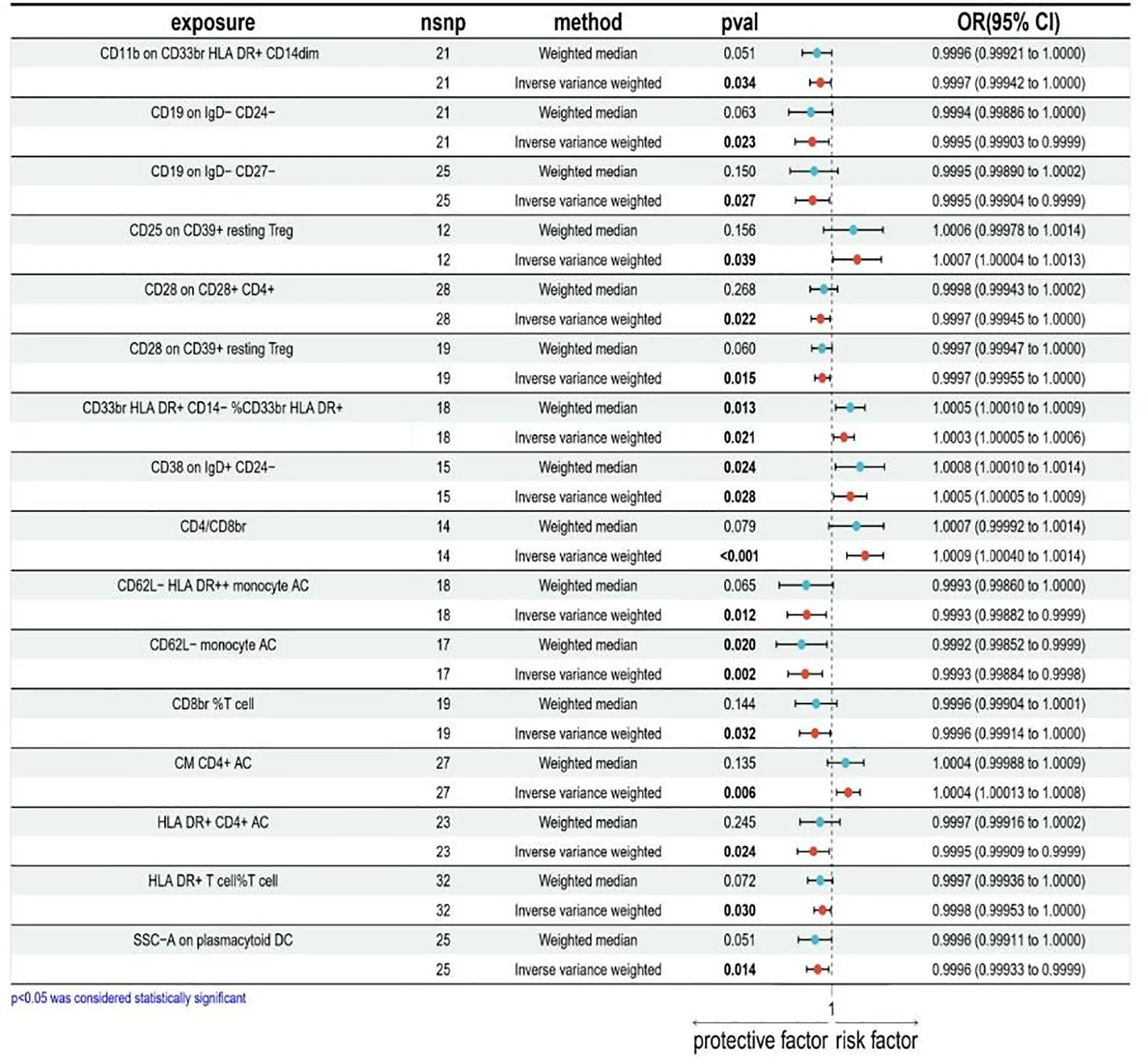
MR analysis of inverse variance weighted and weighted median methods results. CI = confidence interval, MR = Mendelian randomization, OR = odds ratio.

The outcomes from the genetically predicted IVW and weighted median methods for 6 immune cell groups against BCa are illustrated in Figure 3, demonstrating a favorable correlation between the trait of the subsequent 4 immune cells and the onset of BCa (OR > 1, *P* < .05). Myeloid cell team: CD33br HLA DR+ CD14− %CD33br HLA DR+; Maturation stages of T cell team: CM CD4+ AC; TBNK team: CD4/CD8br; B cell team: CD38 on IgD+ CD24−; Treg team: CD25 on CD39+ resting Treg.

However, the remainder BCa incidence decreases by 11 traits (OR < 1, *P* < .05). B cell team: CD19 on IgD− CD24− and CD19 on IgD− CD27−; conventional dendritic cells (cDC) team: CD62L− monocyte AC, CD62L− HLA DR++ monocyte AC and SSC-A on plasmacytoid dendritic cell (DC); TBNK team: CD8br %T cell, HLA DR+ T cell% T cell and HLA DR+ CD4+ AC; Treg team: CD28 on CD28+ CD4+ and CD28 on CD39+ resting Treg; myeloid cell team: CD11b on CD33br HLA DR+ CD14dim. The outcomes of the 5 MR analysis methods are presented in Table S3, Supplemental Digital Content, https://links.lww.com/MD/Q194. These results suggest a complex role of immune cells in BCa development and could help refine therapeutic strategies targeting immune cell modulation.

Sensitivity analyses revealed that none of the top 16 immunocyte phenotypes for MR analysis of BCa were horizontally pleiotropic (*P* > .05 for MR-Egger’s intercept method) or heterogeneous (*P* > .05 for *Q*-test), demonstrating the credibility of causally robust results. Additionally leave-one-out and scatter plots approach both showed trustworthy data (Figs. S1 and S2, Supplemental Digital Content, https://links.lww.com/MD/Q193). The outcomes of the MR sensitivity analysis methods are presented in Table 2. While some cell types have the ability to prevent BCa from starting, others may encourage the development of BCa. These results offer fresh perspectives on the etiology and management of BCa.

**Table 2:** MR sensitivity analysis.

Team	Immune traits	MR-Egger		Inverse variance weighted	
		Intercept	*P* value	*Q*	*P* value
cDC	SSC-A on plasmacytoid DC	0.00006	.306	17.58	.823
B cell	CD19 on IgD− CD24−	0.00009	.261	24.60	.217
TBNK	HLA DR+ T cell%T cell	−0.00008	.138	33.17	.362
Treg	CD28 on CD39+ resting Treg	0.00006	.393	21.87	.238
TBNK	CD8br %T cell	−0.00008	.138	22.04	.230
Treg	CD28 on CD28+ CD4+	−0.00002	.703	21.03	.785
B cell	CD19 on IgD− CD27−	−0.00010	.212	32.03	.126
Maturation stages of T cell	CM CD4+ AC	0.00013	.074	19.11	.832
Treg	CD25 on CD39+ resting Treg	0.00003	.838	14.74	.195
TBNK	HLA DR+ CD4+ AC	−0.00011	.276	19.11	.832
Myeloid cell	CD11b on CD33br HLA DR+ CD14dim	0.00005	.629	113.21	.868
Myeloid cell	CD33br HLA DR+ CD14− % CD33br HLA DR+	−0.00014	.089	15.04	.593
TBNK	CD4/CD8br	0.00011	.240	8.69	.796
cDC	CD62L− HLA DR++ monocyte AC	−0.00005	.642	15.75	.541
B cell	CD19 on IgD− CD24−	0.00009	.261	24.60	.217
cDC	CD62L− monocyte AC	−0.00001	.905	16.03	.451

cDC = conventional dendritic cells, DC = dendritic cells, MR = Mendelian randomization.

In our study, significant associations were observed between various immune cell traits and BCa risk, with OR >1 indicating an increased risk of BCa, and ORs <1 suggesting a protective effect. For instance, the Myeloid cell group (CD33br HLA DR+ CD14− %CD33br HLA DR+) showed a significant positive association with BCa (OR > 1, *P* < .05), indicating a potential role of these cells in promoting cancer development. On the other hand, traits such as CD19 on IgD− CD24− in the B cell group demonstrated an inverse relationship with BCa risk (OR < 1, *P* < .05), suggesting that these cells might offer a protective effect.

The clinical significance of these findings is important, as understanding which immune cell types promote or prevent BCa could provide insights into future immunotherapy strategies and early detection techniques. A larger, more comprehensive study is needed to confirm these associations and determine their precise clinical relevance.

### 3.2. Bladder cancer’ causative relationship to immunophenotypes

We found some encouraging results in the reverse MR analysis. Similarly, no significant findings were seen after adjustment.

## 4. Discussion

Our findings hold significant implications for future research and clinical practices in treating BCa. In this MR study, we identified 16 immune cell types related to 4 immunological signal types (MFI, RC, AC, and MP) that are associated with BCa risk. These mainly include B cells, TBNK cells, myeloid cells, Treg cells, T cell maturation stages, and cDC cells.

Our results show a favorable correlation between the development of BCa and 6 immunocyte morphologies. Notably, CD33br HLA DR+ CD14− %CD33br HLA DR+, CD8br %T cell, HLA DR+ T cell% T cell, HLA DR+ CD4+ AC, CD28 on CD28+ CD4+, CD28 on CD39+ resting Treg, and CD11b on CD33br HLA DR+ CD14 dim have not been directly studied in the context of BCa. The positive correlation between certain immune cell phenotypes, such as CD33br HLA DR+ CD14− %CD33br HLA DR+ and CD8br %T cell, and BCa indicates that these immune cells may play a role in tumor progression. Therapeutic interventions targeting these cells could potentially change the disease course. For example, Liu et al^[26]^ found that the development of primary Sjögren’s syndrome (PSS) is associated with elevated CD33BR HLA DR+ CD14− %CD33BR HLA DR+ in myeloid cells. CD25, a part of the interleukin-2 (IL-2) receptor, is expressed on both immune and non - immune cells. It is highly expressed on regulatory T cells (Tregs), which are known to promote tumor growth.^[27]^ Research has shown that anti-CD25 antibodies can reduce Treg counts, exerting an antitumor immunological effect. Wojciech et al demonstrated that regulatory T cells (Tregs; CD4+ CD25+ FoxP3+) promote immune tolerance and contribute to the development of BCa. They also found that Treg frequencies in the later stages of tumor growth are linked to a reduced antitumor response, serving as a novel and important prognostic factor in BCa.^[28]^ IgD+ B cells, a subpopulation of B cells, express IgD on their surface to initiate immune responses. IgD can coexist with other immunoglobulins on B cells to jointly regulate immune responses.^[29,30]^ CD4+ T cells can not only transform myeloid cells into IFNγ-induced antigen - presenting cells but also reprogram them into tumoricidal effectors expressing iNOS, which can eliminate tumors that evade the immune system.^[31]^ The exhaustion of cytotoxic CD4+ T cells and CD8+ T cells is one of the mechanisms of tumor immune evasion.^[32]^ The positive link between the development of BCa and CM CD4+ AC and CD4/CD8br indicates that these immune cell phenotypes may be involved in tumor-promoting processes. The identification of these cell types provides new perspectives on the complex role of the immune system in BCa progression. Rituximab treatment in B-cell non-Hodgkin’s lymphoma (B-NHL) patients significantly reduces peripheral blood CD19+/CD20+ B-cell counts. Notably, residual CD19+ B-cells detected 6 months posttreatment exhibit high CD38/CD24 expression and display a naïve B-cell phenotype (IgD+ CD27−). These findings suggest that IgD+ B-cells may contribute to B-NHL pathogenesis via specific pathways, identifying them as a potential risk factor for B-NHL.^[33]^ While direct evidence linking IgD+ B-cells to BCa remains lacking, this mechanism provides a framework for investigating their role in BCa.

Leonie et al demonstrated that IgD− CD11c+ CD21− low and IgD− CD24+ CD21+ high B-cells are significantly reduced in patients with autoantibody-positive neurologic irAEs (irAE-n), implicating IgD− B-cells in the pathogenesis of irAE-n and identifying them as a protective factor.^[34]^ Furthermore, their findings suggest a potential negative association between BCa development and: IgD− CD24− % B-cells, (2) CD19+ (IgD− CD24−), and (3) CD19+ (IgD− CD27−), highlighting the complex role of B-cell subsets in cancer immunity.

DCs, critical for initiating adaptive immune responses, regulate *T*-helper cell polarization and immune activation.^[35,36]^ Altered DC profiles – including migration, tissue distribution, antigen presentation, and cytokine secretion – contribute to autoimmune disease pathogenesis.^[35,36]^ Vaccine-based strategies using DCs have emerged as promising immunotherapies for cancer. Zhang et al developed a DC-based vaccine that inhibits bladder tumor growth in vivo and enhances chemotherapy efficacy, demonstrating the antitumor potential of antigen-loaded mature DCs.^[37]^ Our MR analysis corroborates these findings, revealing significant negative associations between BCa risk and 3 DC-related phenotypes: CD62L− monocytic ACs, CD62L− HLA-DR++ monocytic ACs, and SSC-A in plasmacytoid DCs (pDCs) within the cDC population. CD62L (L-selectin), a cell adhesion molecule mediating leukocyte rolling/adhesion/migration via endothelial ligand binding,^[38]^ enhances anti-tumor immunity by promoting DC chemotaxis. While the therapeutic role of pDCs in the tumor microenvironment remains underexplored.^[19]^ Masanori et al observed elevated pDC levels in triple-negative breast cancer, correlating with high expression of immune checkpoint markers and abundant antitumor immune cells. Notably, pDC levels showed stronger associations with immune infiltration and patient survival than conventional DCs (cDCs) in triple-negative breast cancer,^[39]^ suggesting their potential as BCa immunotherapy targets.

In the clinic, these findings could guide the development of personalized immunotherapy treatments targeting specific immune cell phenotypes. This would enhance the effectiveness of existing therapies and offer hope to patients who do not respond to current treatments. Bakouny et al showed that pre-cytopenic nephrectomy was associated with a significant overall survival benefit via immune checkpoint inhibitors, suggesting that immune checkpoint inhibitor immunomodulatory strategies can be tailored to target these newly identified immune cells, thereby suppressing tumorigenesis.

The strengths of our study lie in its use of MR methods to explore the connection between immune cell morphologies and BCa. By closely examining horizontal pleiotropy, we reduced the influence of reverse causality and confounding variables. Moreover, we discovered immune cell morphologies that have not been well-studied but are strongly associated with BCa, offering fresh perspectives on potential immunotherapy targets.

## 5. Limitations

Our study has several limitations. First, although our research includes 731 types of immune cell morphologies and 1279 BCa patients, the relatively small sample size may limit the statistical significance and generalizability of the results. Second, GWAS data are limited to adults of predominantly European ancestry, which precludes stratification by sex or age, potentially affecting the accuracy and broader applicability of the findings. Third, using a lower threshold (*P* < 1.0 × 10^−5^) when selecting IVs may lead to false positives or miss important genetic variants associated with immune cell characteristics. Fourth, while our study indicates a limited association between immune cells and the incidence of BCa, an important issue is that the study did not include independent validation studies to confirm the findings. The lack of additional validation data may limit the robustness and credibility of the conclusions. Fifth, our investigation focused on specific immune cell types and morphologies, but there may be other important immune subtypes that were not captured in the analysis. Additionally, immune cell phenotyping methods can vary between studies, which may limit the generalizability of our findings. Future research that includes a broader range of immune cell types and standardized phenotyping methods would help to validate our results and expand the understanding of immune cell roles in BCa. Future studies should take these limitations into account in order to refine the findings of this paper and help translate them into clinical practice. To further explore our findings and investigate potential mechanisms, future work should aim to overcome these challenges through the inclusion of larger, more diverse populations, better phenotypic characterization, and experimental validation of the mechanisms by which immune cells are associated with BCa risk.

## 6. Conclusions

Through a comprehensive bidirectional MR analysis, we were able to reveal a complicated causal link between several immunological phenotypes and BCa, focusing on the intricate network of interactions between the immune system and BCa. The results of this study provide fresh viewpoints and resources for investigating immunotherapeutic targets and BCa preventive tactics. While our study offers valuable insights into the immune cell phenotypes associated with BCa risk, future research should aim to further investigate the mechanisms underlying these associations. Longitudinal studies are needed to validate the causal relationships between immune cell phenotypes and BCa in diverse populations. Additionally, clinical trials focusing on immune modulation strategies targeting these immune cell subsets could provide critical insights into new immunotherapeutic options for BCa patients. Finally, expanding the scope of immune cell profiling and incorporating advanced techniques, such as single-cell RNA sequencing, could uncover more detailed immune signatures that might play a role in BCa pathogenesis and progression.

## Acknowledgments

We appreciate the data sharing provided by GWAS database contributors.

## Author contributions

**Conceptualization:** Fuwen Luo.

**Data curation:** Chunyan Hu, Yuhang Lin, Junyang Li, Fuwen Luo.

**Formal analysis:** Chunyan Hu, Yuhang Lin.

**Methodology:** Chunyan Hu, Junyang Li.

**Software:** Xiaole Zhu.

**Visualization:** Xiaodong Yu.

**Writing** – **original draft:** Anneng Hu, Xiaole Zhu.

**Writing** – **review & editing:** Anneng Hu.

## Supplementary Material
