# Supplementary material for: Causal relationship between immune cells and bladder cancer: A bidirectional Mendelian randomization study
Source: Medicine (Baltimore). 2025 Sep 26;104(39):e44268. doi: 10.1097/MD.0000000000044268 (PMC13593239; doi:10.1097/MD.0000000000044268)
Supplement: Supplementary file 1 [file medi-104-e44268-s003.pdf]

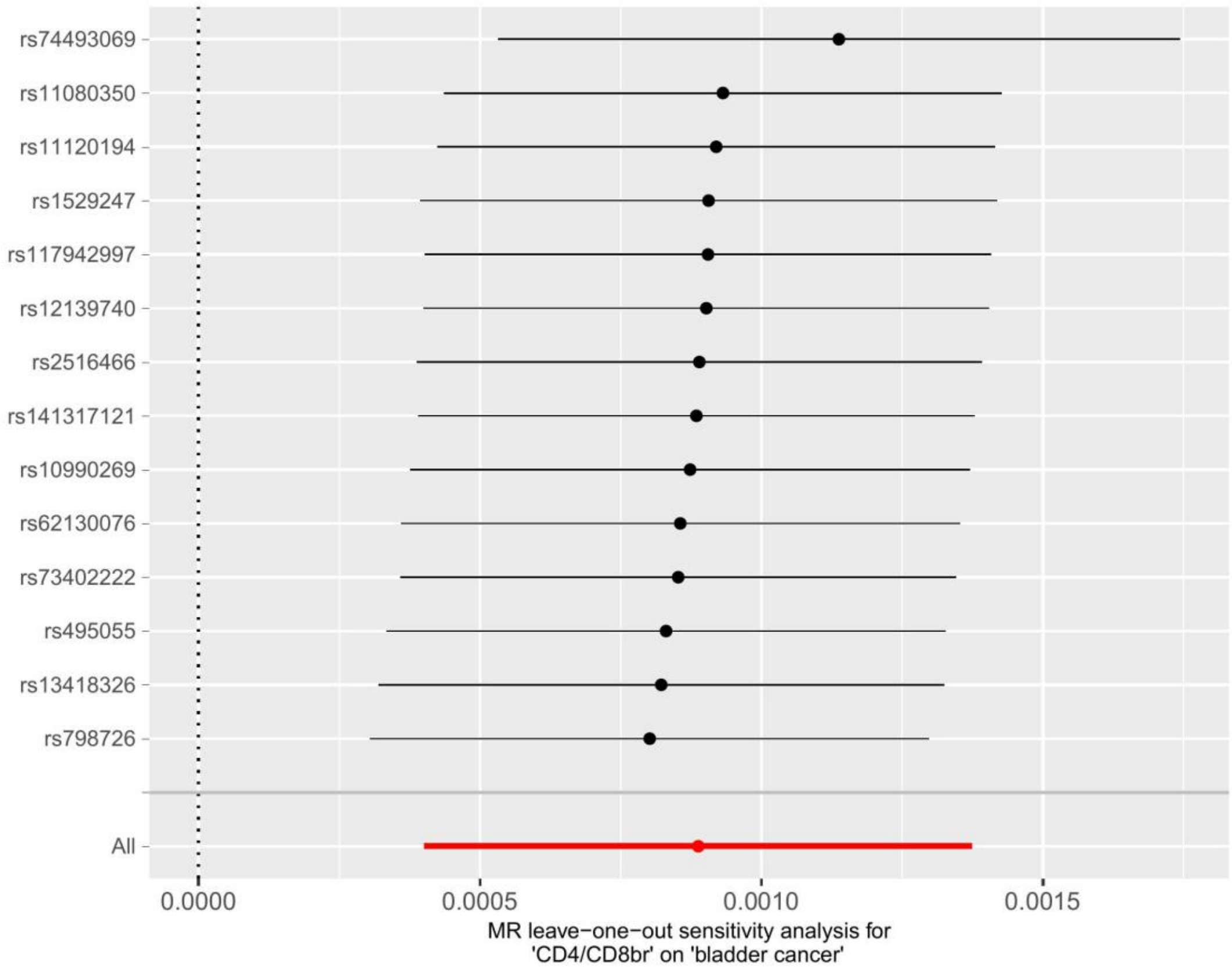
rs74493069
rs11080350
rs11120194
rs1529247
rs117942997
rs12139740
rs2516466
rs141317121
rs10990269
rs62130076
rs73402222
rs495055
rs13418326
rs798726
All
0.0000
0.0005
0.0010
0.0015
MR leave−one−out sensitivity analysis for
'CD4/CD8br' on 'bladder cancer'

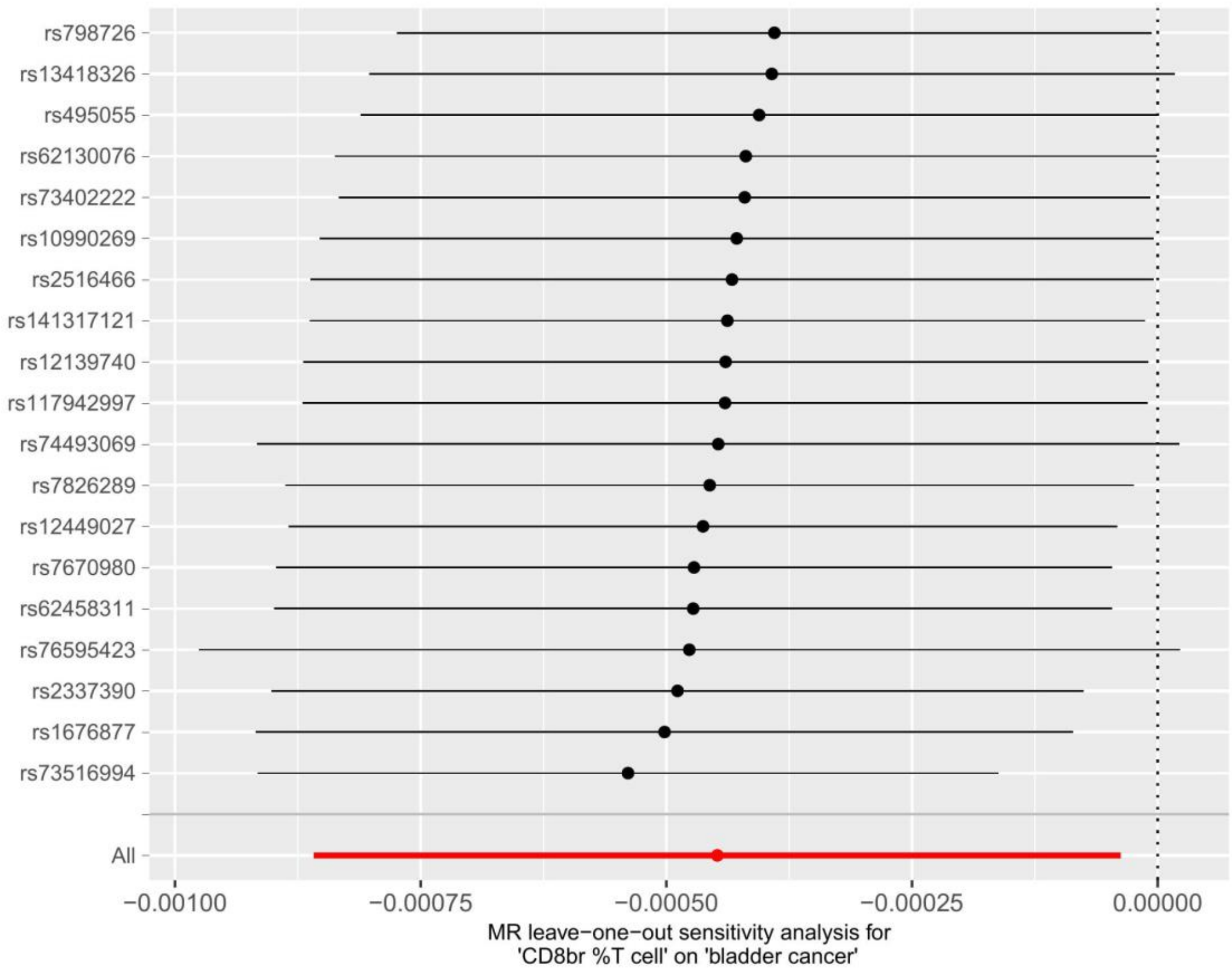
rs798726
rs13418326
rs495055
rs62130076
rs73402222
rs10990269
rs2516466
rs141317121
rs12139740
rs117942997
rs74493069
rs7826289
rs12449027
rs7670980
rs62458311
rs76595423
rs2337390
rs1676877
rs73516994
All
−0.00100
−0.00075
−0.00050
−0.00025
0.00000
MR leave−one−out sensitivity analysis for
'CD8br %T cell' on 'bladder cancer'

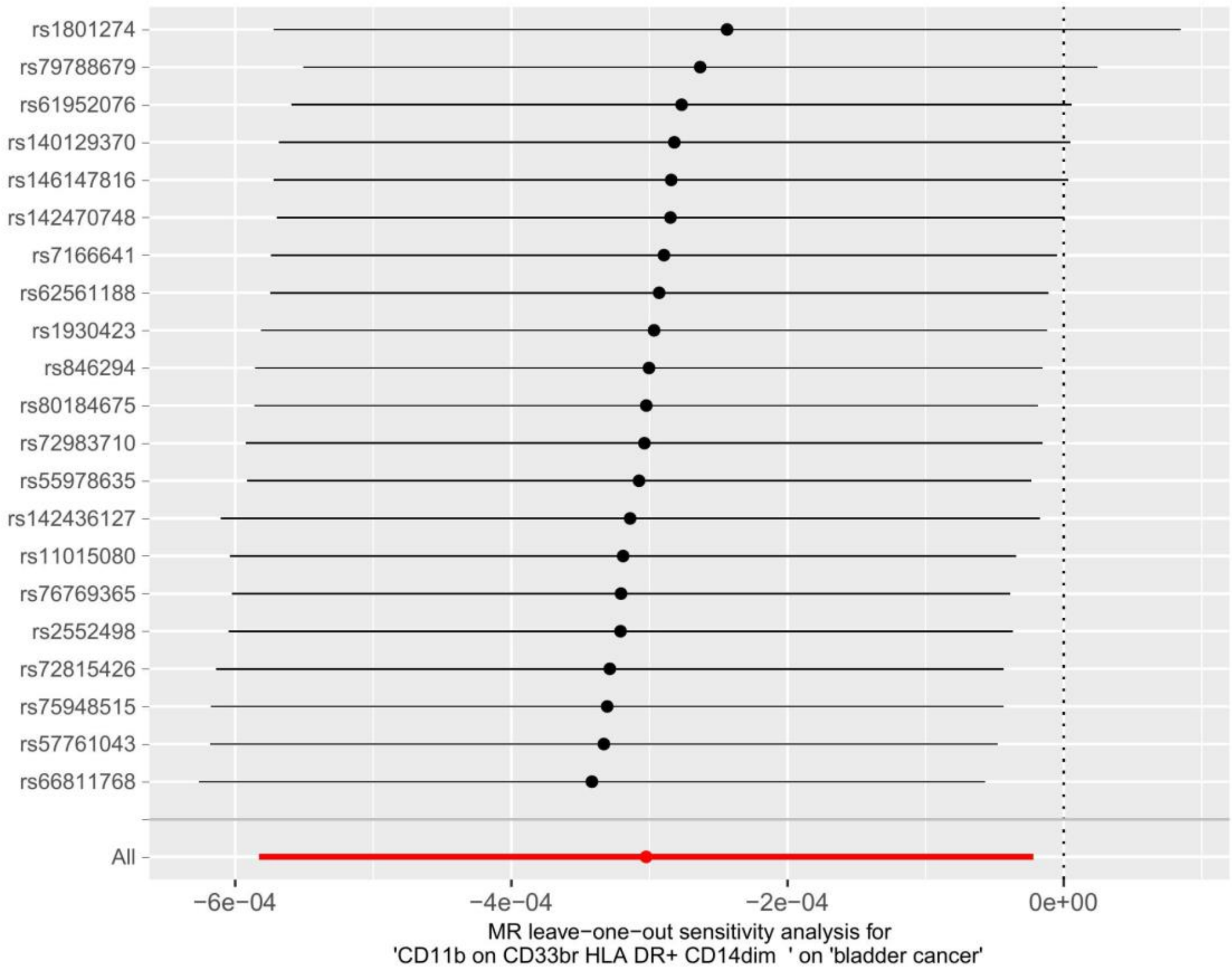
rs1801274
rs79788679
rs61952076
rs140129370
rs146147816
rs142470748
rs7166641
rs62561188
rs1930423
rs846294
rs80184675
rs72983710
rs55978635
rs142436127
rs11015080
rs76769365
rs2552498
rs72815426
rs75948515
rs57761043
rs66811768
All
−6e−04
−4e−04
−2e−04
0e+00
MR leave−one−out sensitivity analysis for
'CD11b on CD33br HLA DR+ CD14dim ' on 'bladder cancer'

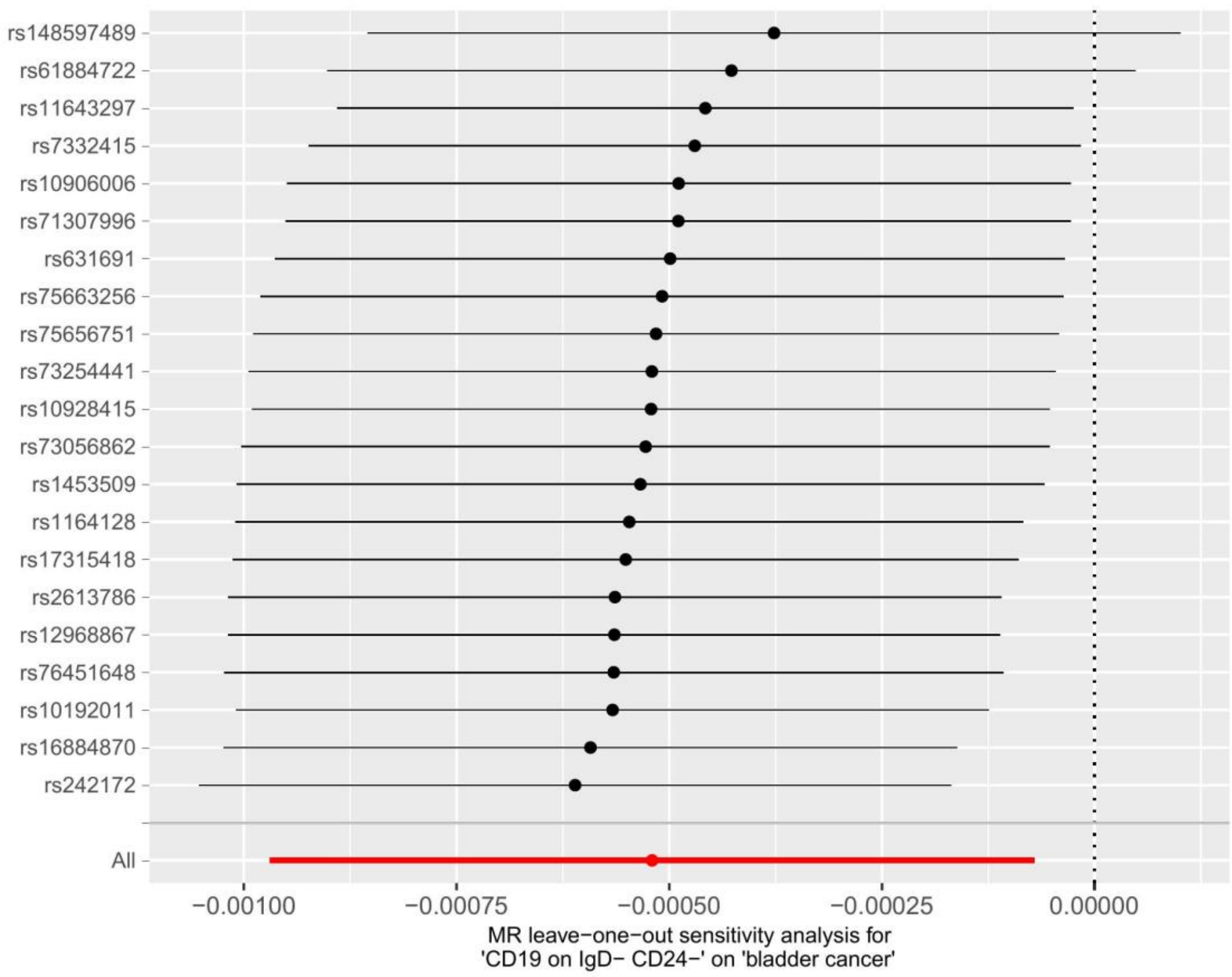
rs148597489
rs61884722
rs11643297
rs7332415
rs10906006
rs71307996
rs631691
rs75663256
rs75656751
rs73254441
rs10928415
rs73056862
rs1453509
rs1164128
rs17315418
rs2613786
rs12968867
rs76451648
rs10192011
rs16884870
rs242172
All
−0.00100
−0.00075
−0.00050
−0.00025
0.00000
MR leave−one−out sensitivity analysis for
'CD19 on IgD− CD24−' on 'bladder cancer'

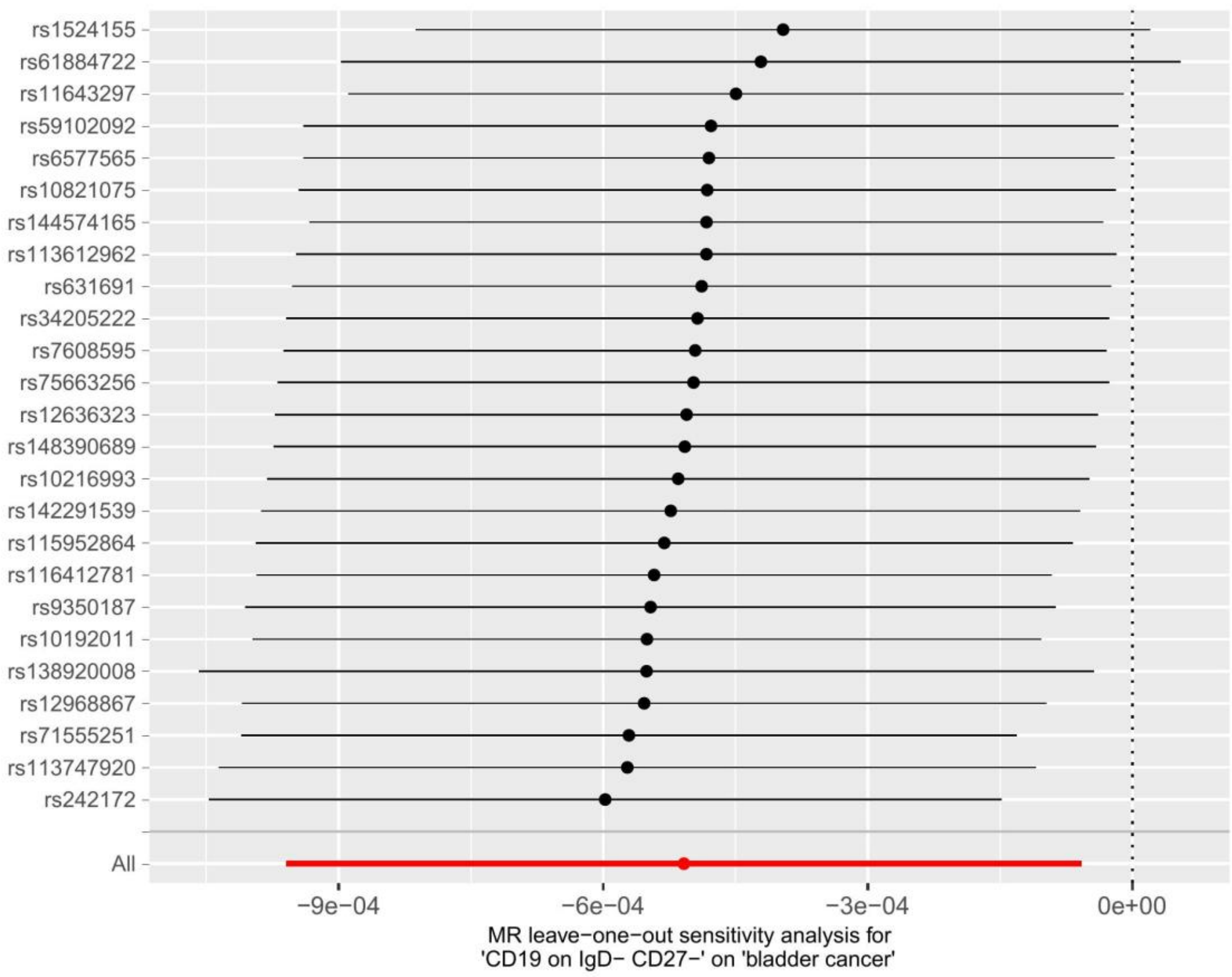
rs1524155
rs61884722
rs11643297
rs59102092
rs6577565
rs10821075
rs144574165
rs113612962
rs631691
rs34205222
rs7608595
rs75663256
rs12636323
rs148390689
rs10216993
rs142291539
rs115952864
rs116412781
rs9350187
rs10192011
rs138920008
rs12968867
rs71555251
rs113747920
rs242172
All
−9e−04
−6e−04
−3e−04
0e+00
MR leave−one−out sensitivity analysis for
'CD19 on IgD− CD27−' on 'bladder cancer'

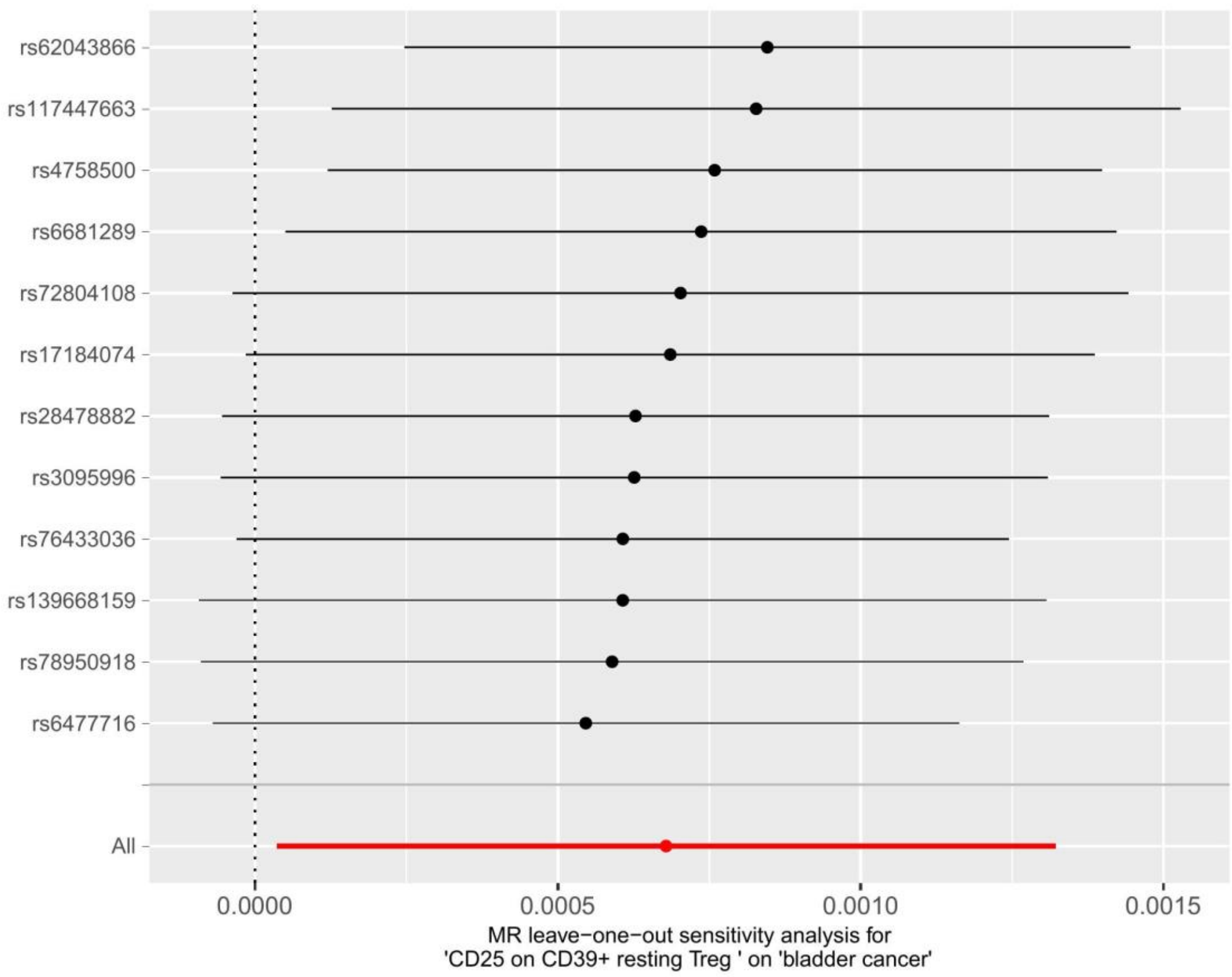
rs62043866
rs117447663
rs4758500
rs6681289
rs72804108
rs17184074
rs28478882
rs3095996
rs76433036
rs139668159
rs78950918
rs6477716
All
0.0000
0.0005
0.0010
0.0015
MR leave−one−out sensitivity analysis for
'CD25 on CD39+ resting Treg ' on 'bladder cancer'

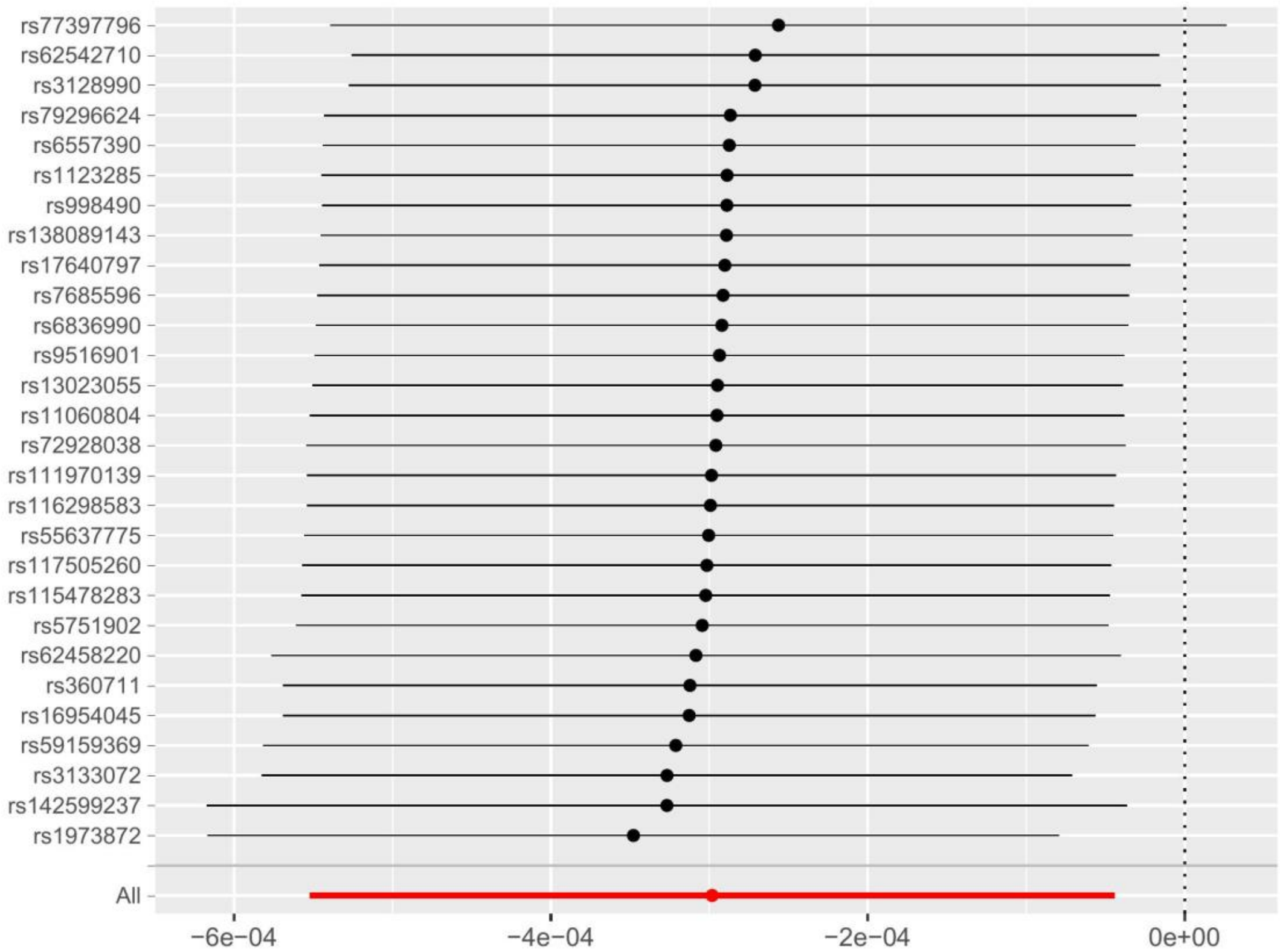

MR leave−one−out sensitivity analysis for
'CD28 on CD28+ CD4+ ' on 'bladder cancer'

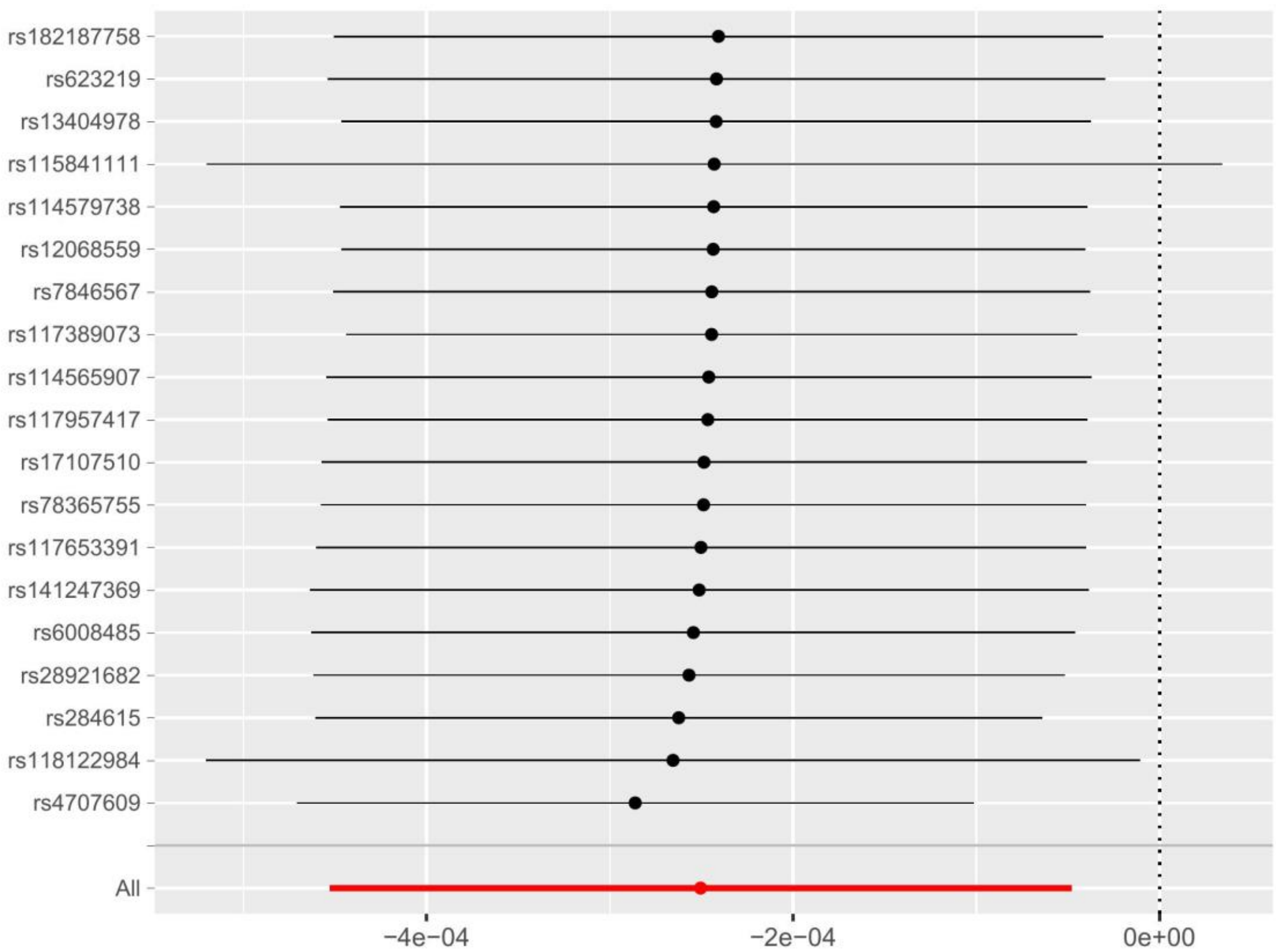

MR leave−one−out sensitivity analysis for
'CD28 on CD39+ resting Treg ' on 'bladder cancer'

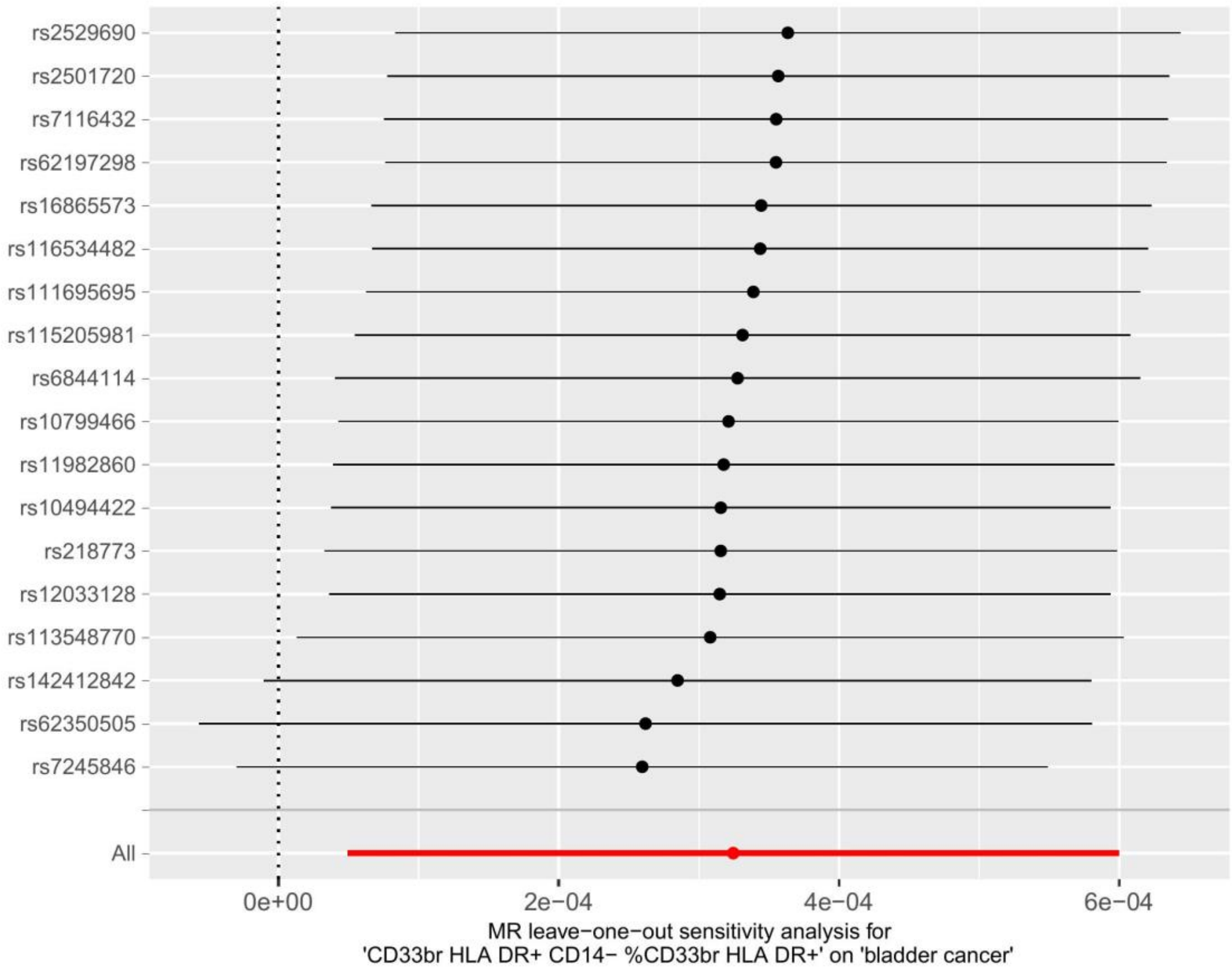
rs2529690
rs2501720
rs7116432
rs62197298
rs16865573
rs116534482
rs111695695
rs115205981
rs6844114
rs10799466
rs11982860
rs10494422
rs218773
rs12033128
rs113548770
rs142412842
rs62350505
rs7245846
All
0e+00
2e-04
4e-04
6e-04
MR leave-one-out sensitivity analysis for
'CD33br HLA DR+ CD14- %CD33br HLA DR+' on 'bladder cancer'

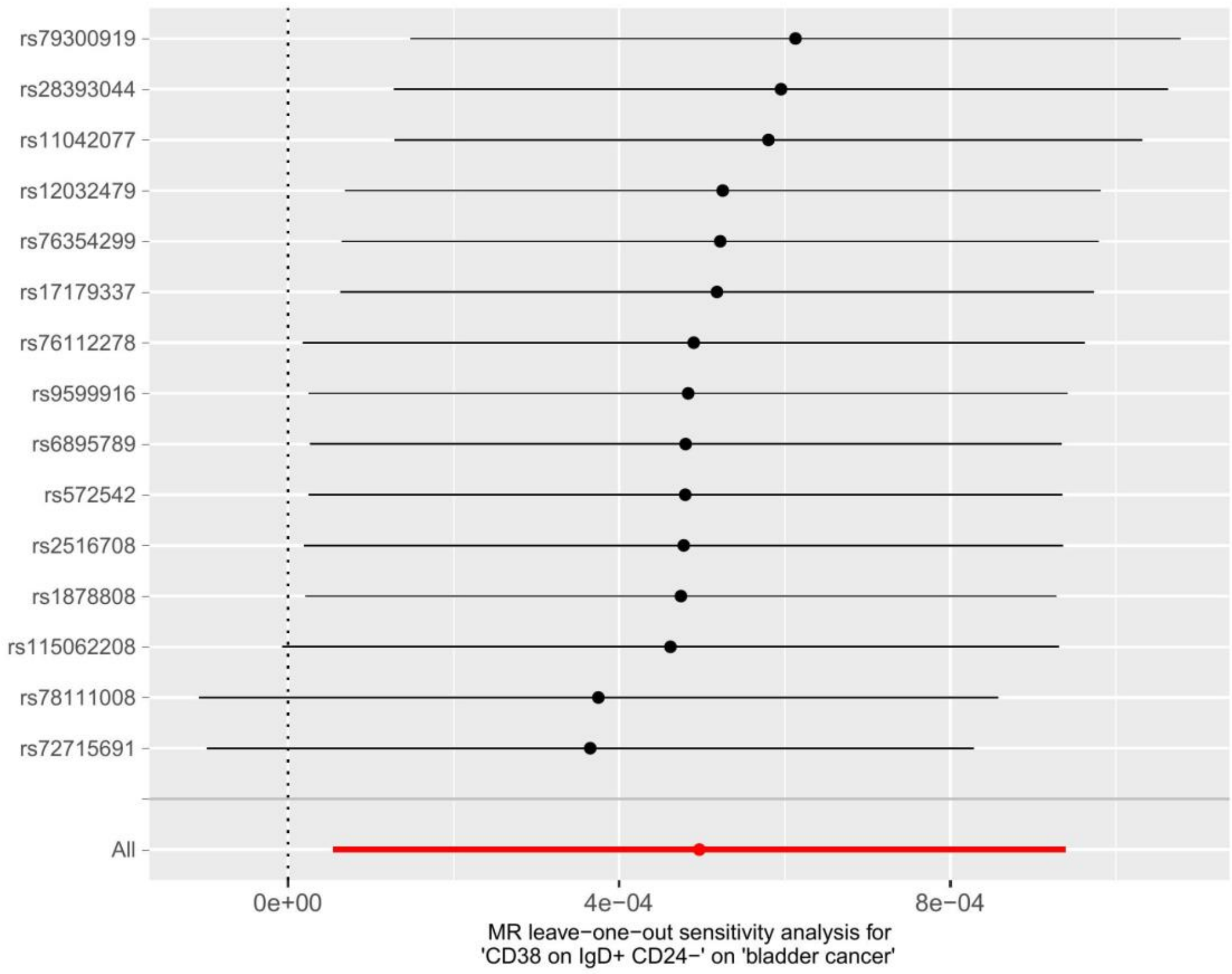
rs79300919
rs28393044
rs11042077
rs12032479
rs76354299
rs17179337
rs76112278
rs9599916
rs6895789
rs572542
rs2516708
rs1878808
rs115062208
rs78111008
rs72715691
All
0e+00
4e-04
8e-04
MR leave-one-out sensitivity analysis for
'CD38 on IgD+ CD24-' on 'bladder cancer'

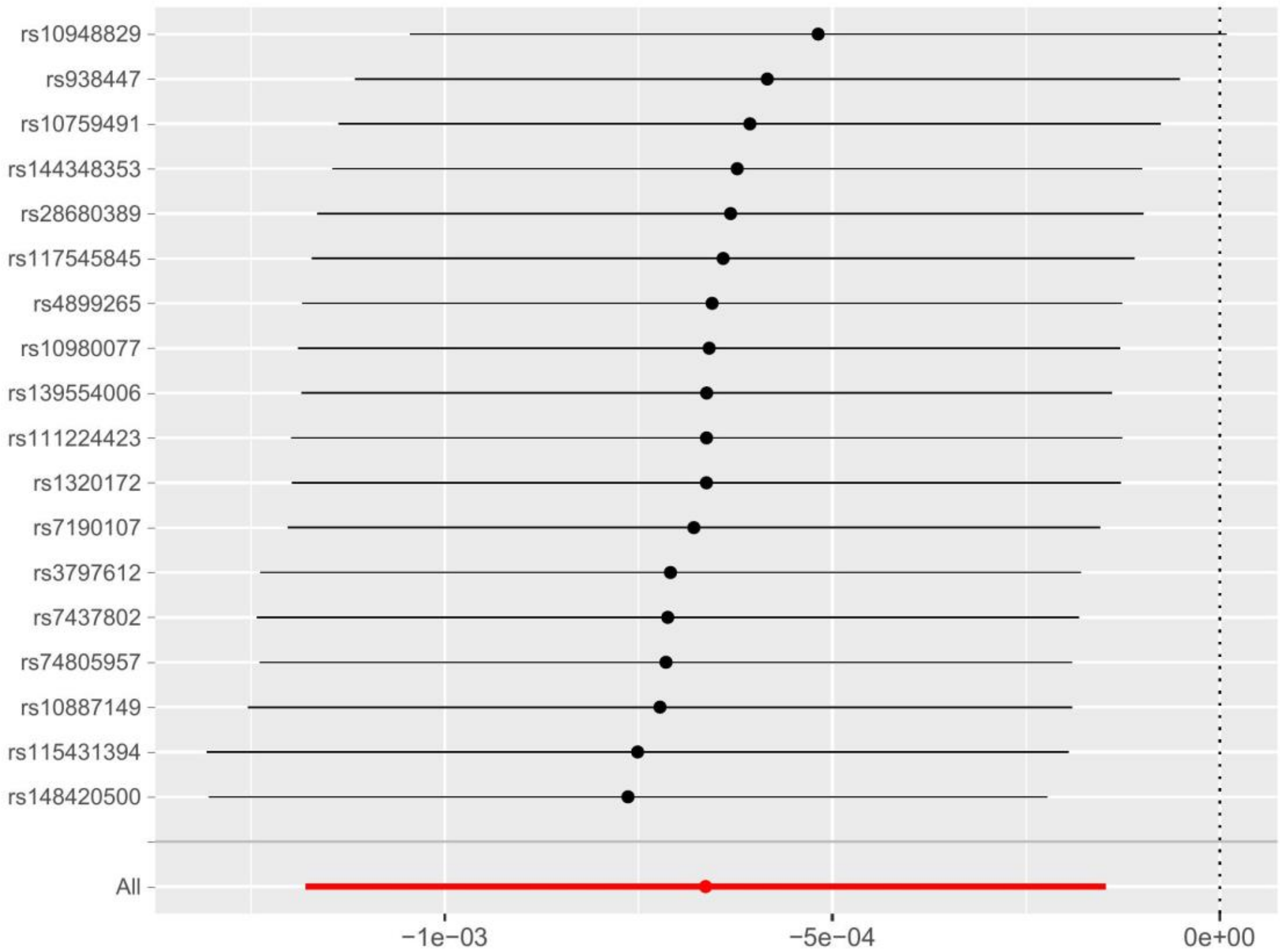

MR leave−one−out sensitivity analysis for
'CD62L− HLA DR++ monocyte AC' on 'bladder cancer'

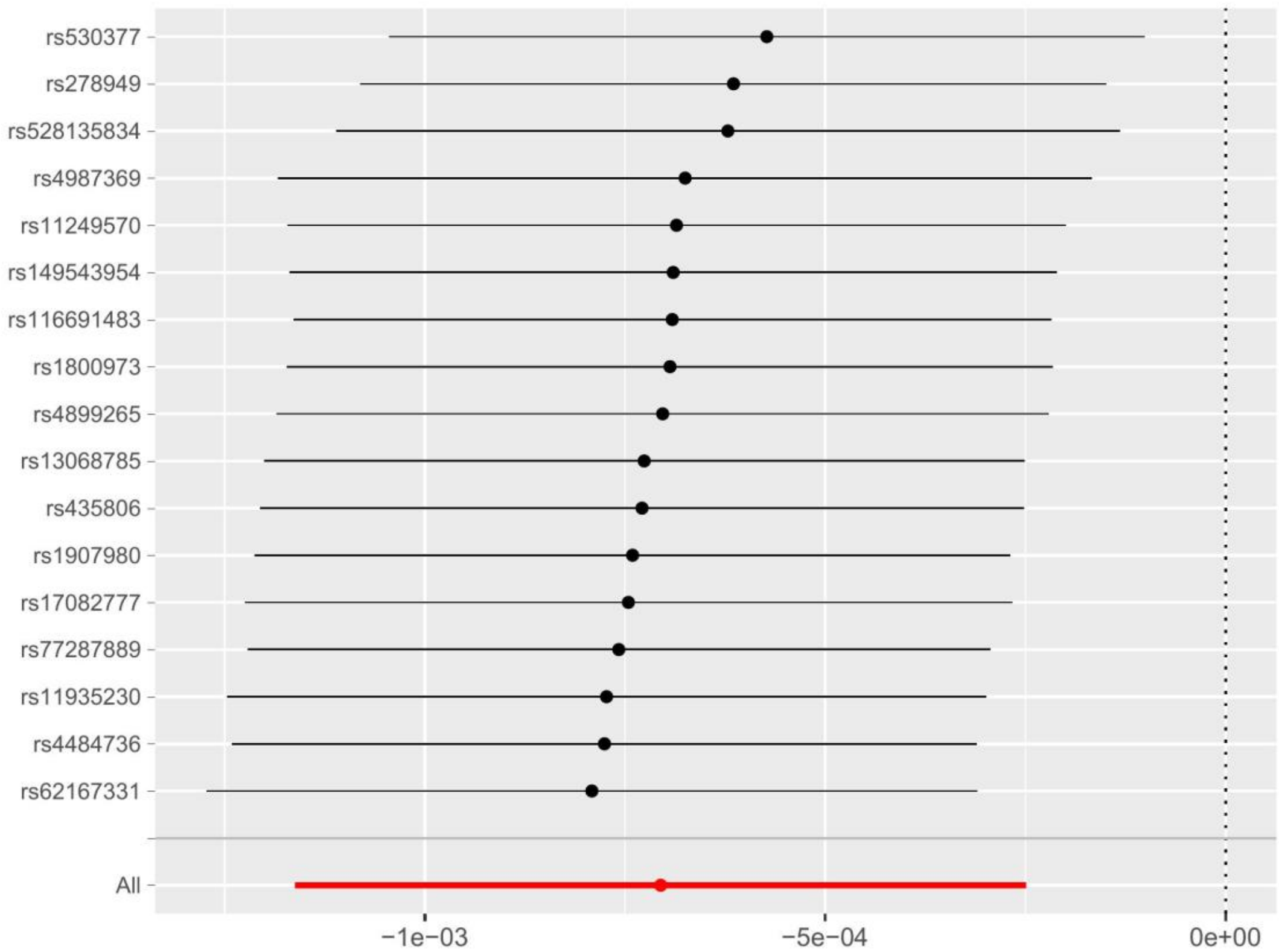

MR leave−one−out sensitivity analysis for
'CD62L− monocyte AC' on 'bladder cancer'

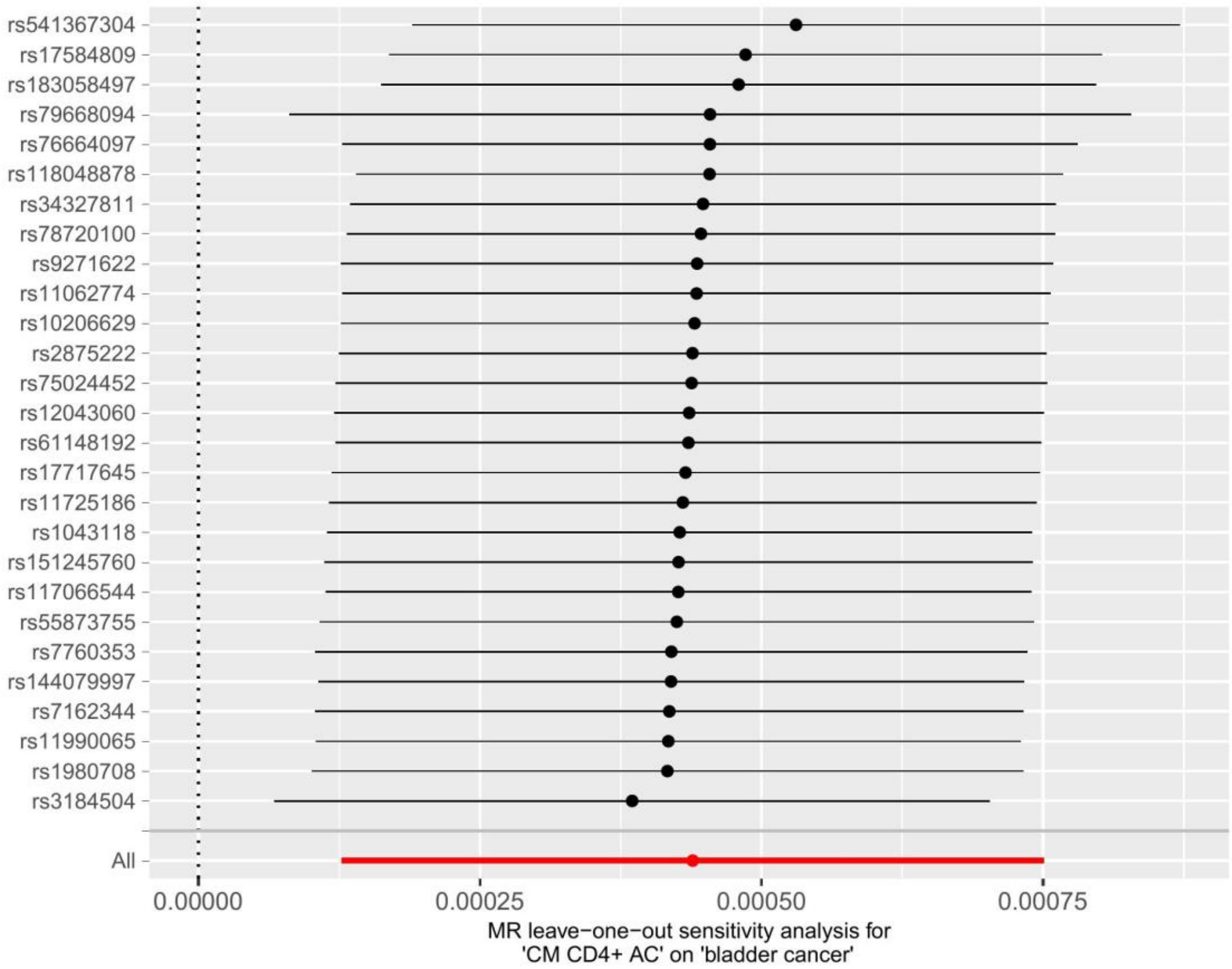

rs541367304
rs17584809
rs183058497
rs79668094
rs76664097
rs118048878
rs34327811
rs78720100
rs9271622
rs11062774
rs10206629
rs2875222
rs75024452
rs12043060
rs61148192
rs17717645
rs11725186
rs1043118
rs151245760
rs117066544
rs55873755
rs7760353
rs144079997
rs7162344
rs11990065
rs1980708
rs3184504
All
0.00000
0.00025
0.00050
0.00075
MR leave−one−out sensitivity analysis for
'CM CD4+ AC' on 'bladder cancer'

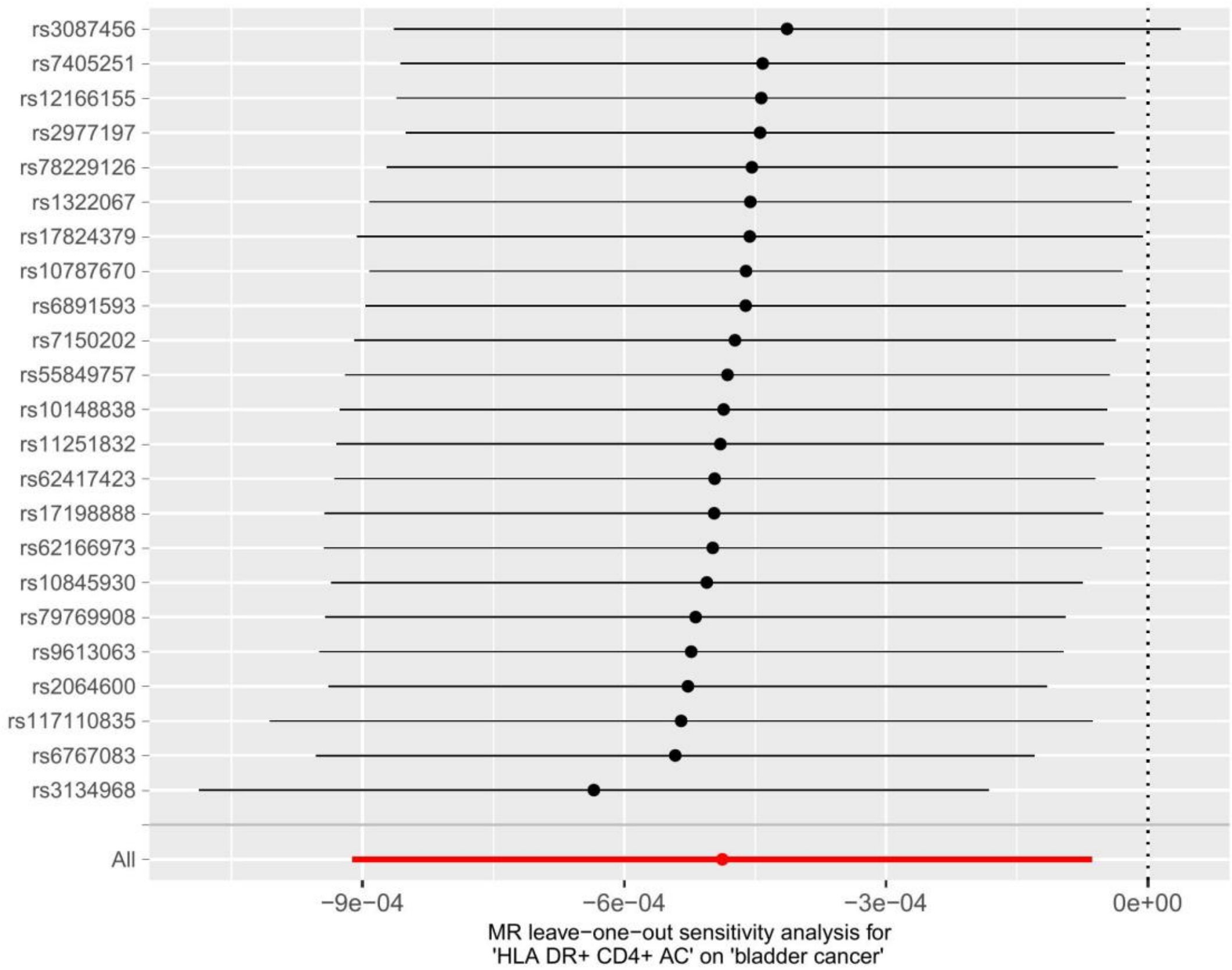

rs3087456
rs7405251
rs12166155
rs2977197
rs78229126
rs1322067
rs17824379
rs10787670
rs6891593
rs7150202
rs55849757
rs10148838
rs11251832
rs62417423
rs17198888
rs62166973
rs10845930
rs79769908
rs9613063
rs2064600
rs117110835
rs6767083
rs3134968
All
−9e−04
−6e−04
−3e−04
0e+00
MR leave−one−out sensitivity analysis for
'HLA DR+ CD4+ AC' on 'bladder cancer'

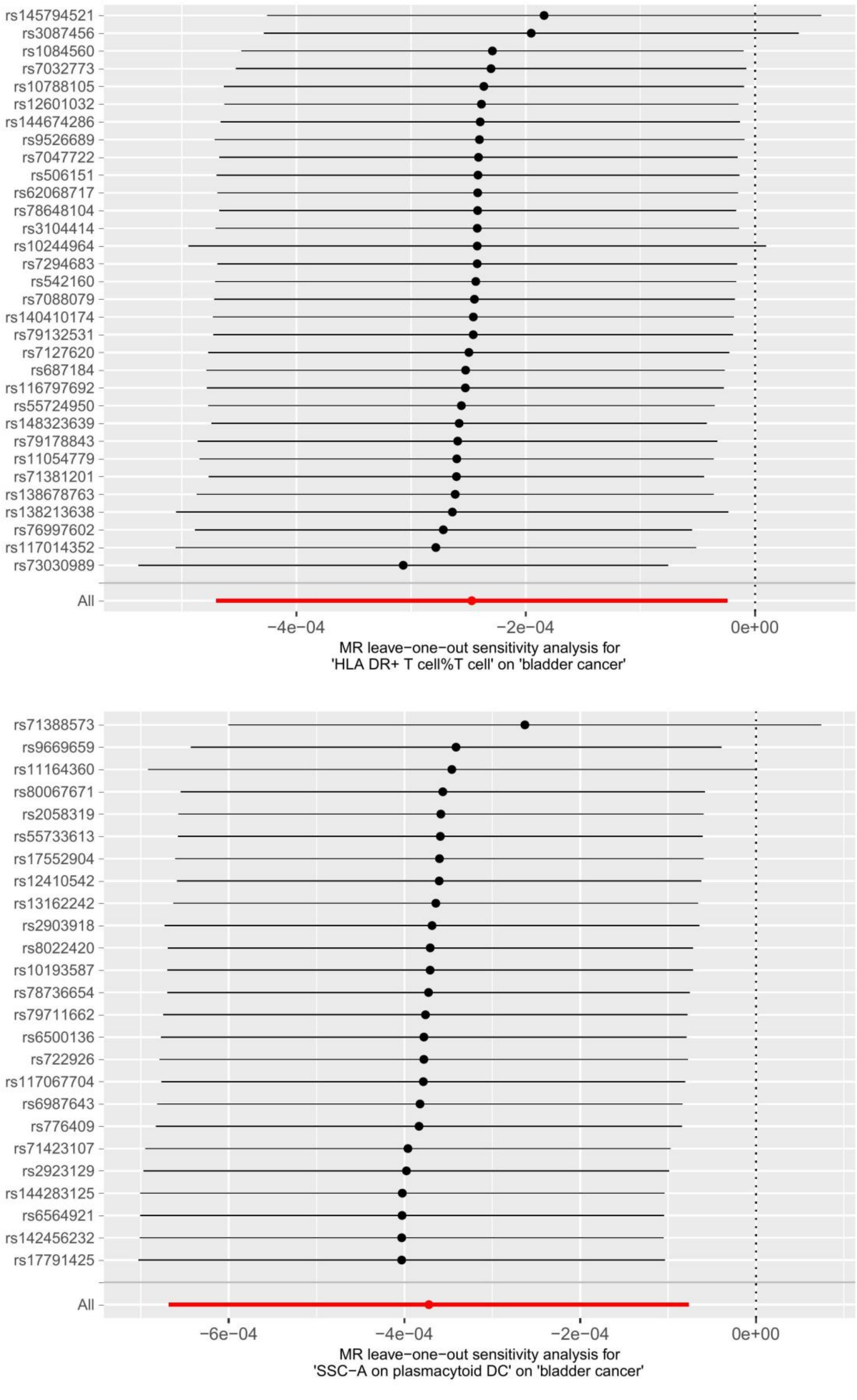

Supplementary Figure 1 A leave-one-out analysis of 16 immune cells and bladder cancer

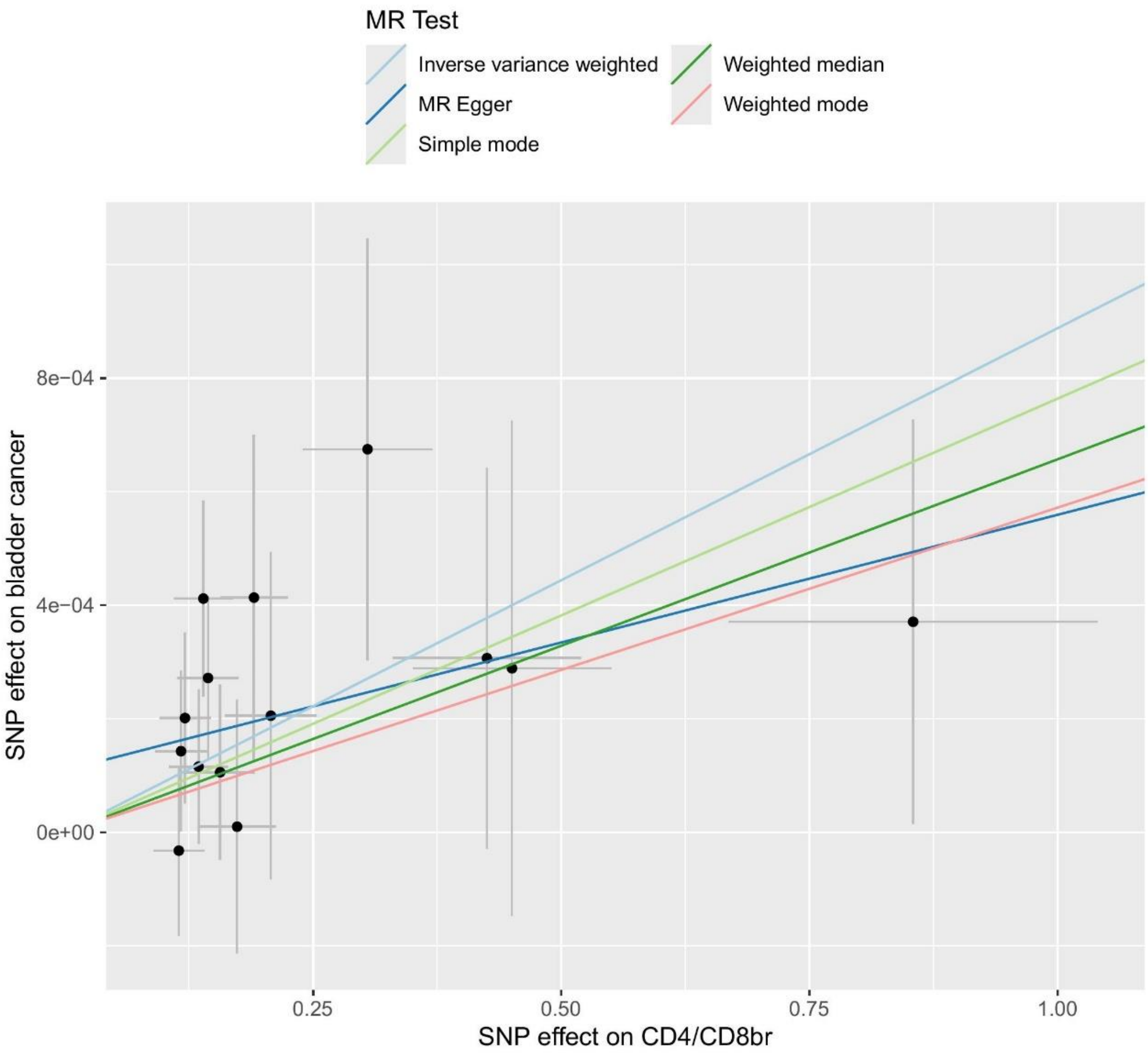
MR Test
Inverse variance weighted
MR Egger
Simple mode
Weighted median
Weighted mode
8e−04
4e−04
0e+00
SNP effect on bladder cancer
0.25
0.50
0.75
1.00
SNP effect on CD4/CD8br

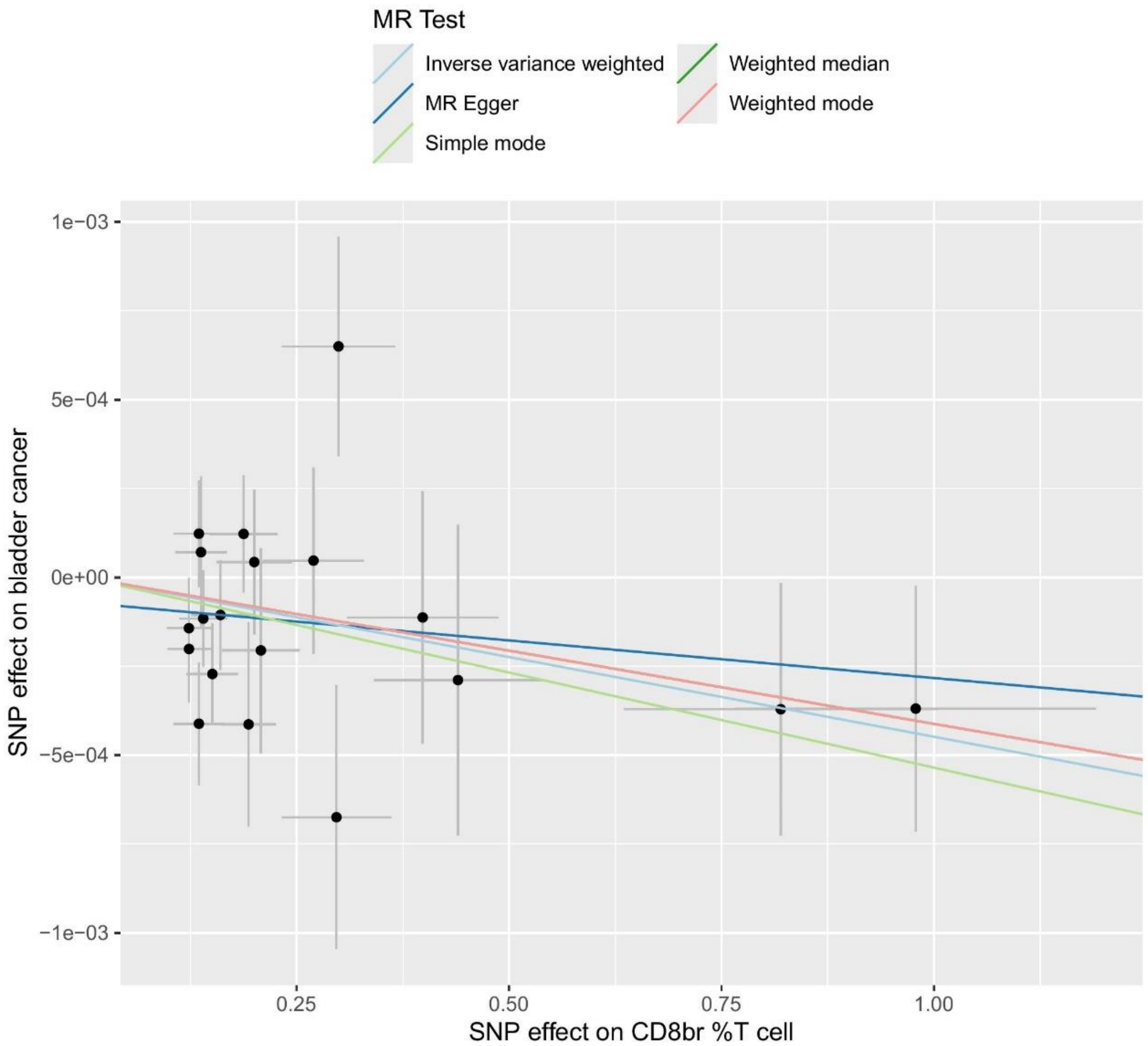
MR Test
Inverse variance weighted
MR Egger
Simple mode
Weighted median
Weighted mode
1e−03
5e−04
0e+00
−5e−04
−1e−03
SNP effect on bladder cancer
0.25
0.50
0.75
1.00
SNP effect on CD8br %T cell

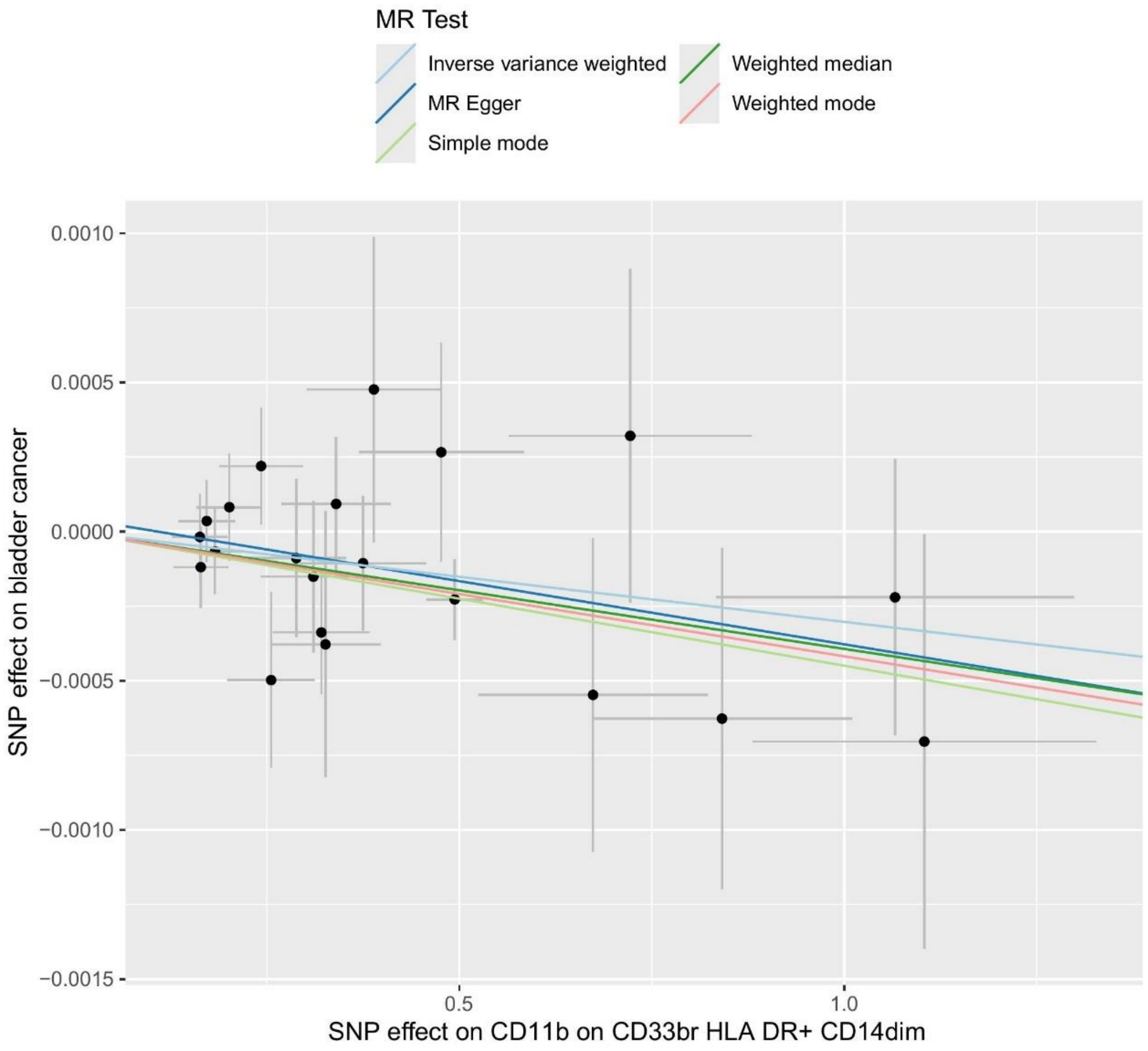
MR Test
Inverse variance weighted
MR Egger
Simple mode
Weighted median
Weighted mode
0.0010
0.0005
0.0000
−0.0005
−0.0010
−0.0015
SNP effect on bladder cancer
0.5
1.0
SNP effect on CD11b on CD33br HLA DR+ CD14dim

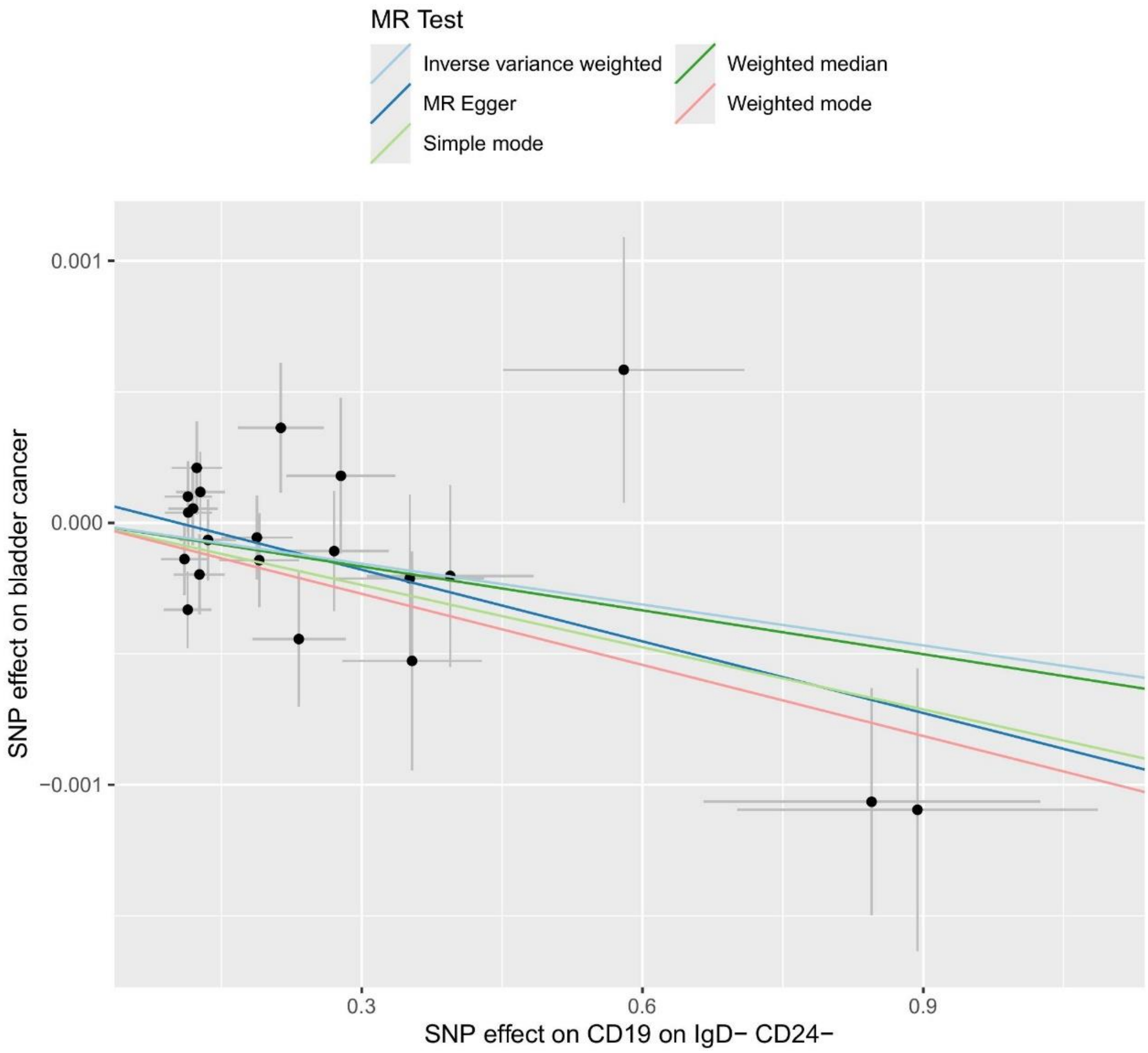
MR Test
Inverse variance weighted
MR Egger
Simple mode
Weighted median
Weighted mode
0.001
0.000
−0.001
SNP effect on bladder cancer
0.3
0.6
0.9
SNP effect on CD19 on IgD− CD24−

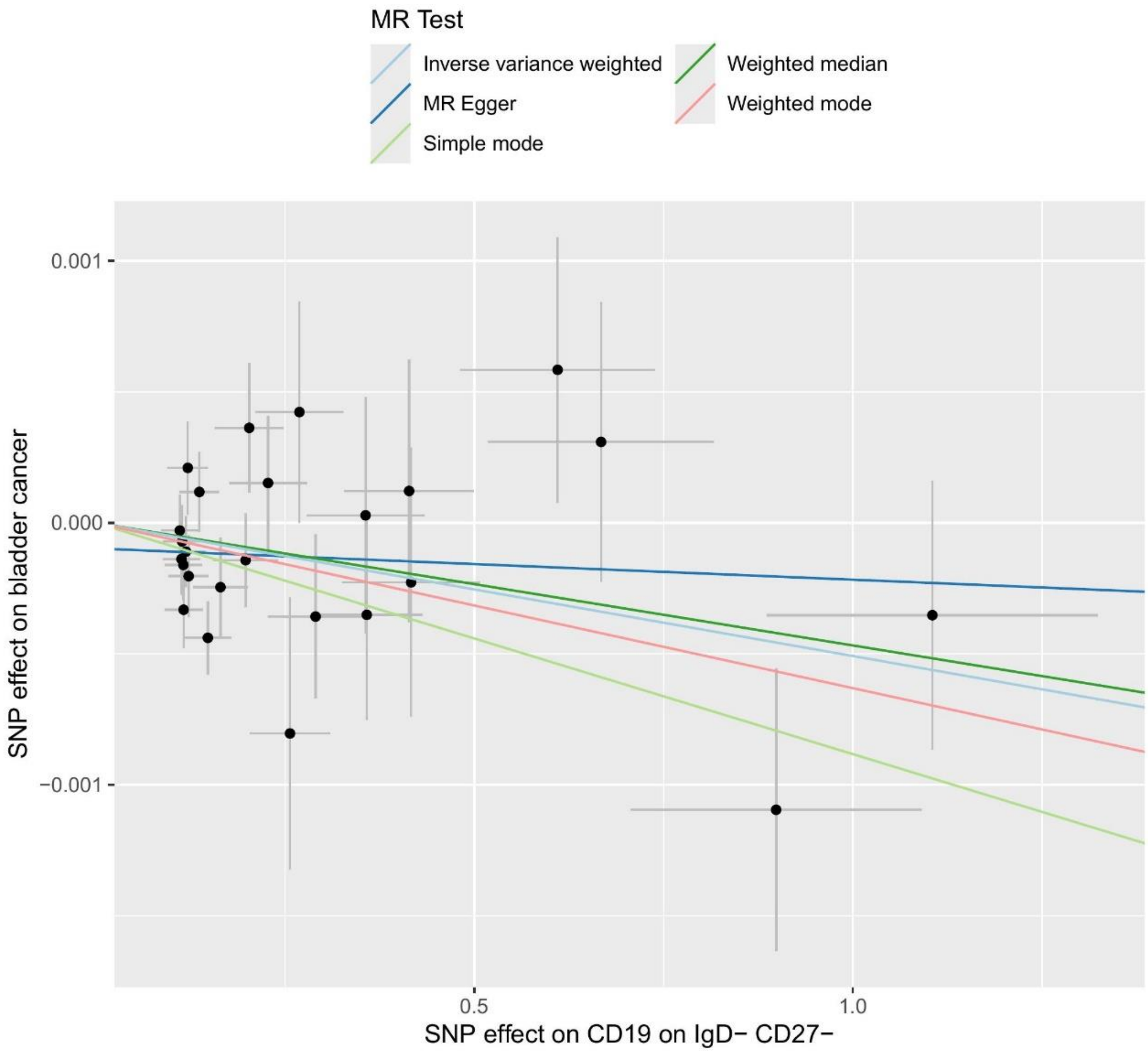

MR Test
Inverse variance weighted
MR Egger
Simple mode
Weighted median
Weighted mode
0.001
0.000
−0.001
0.5
1.0
SNP effect on bladder cancer
SNP effect on CD19 on IgD− CD27−

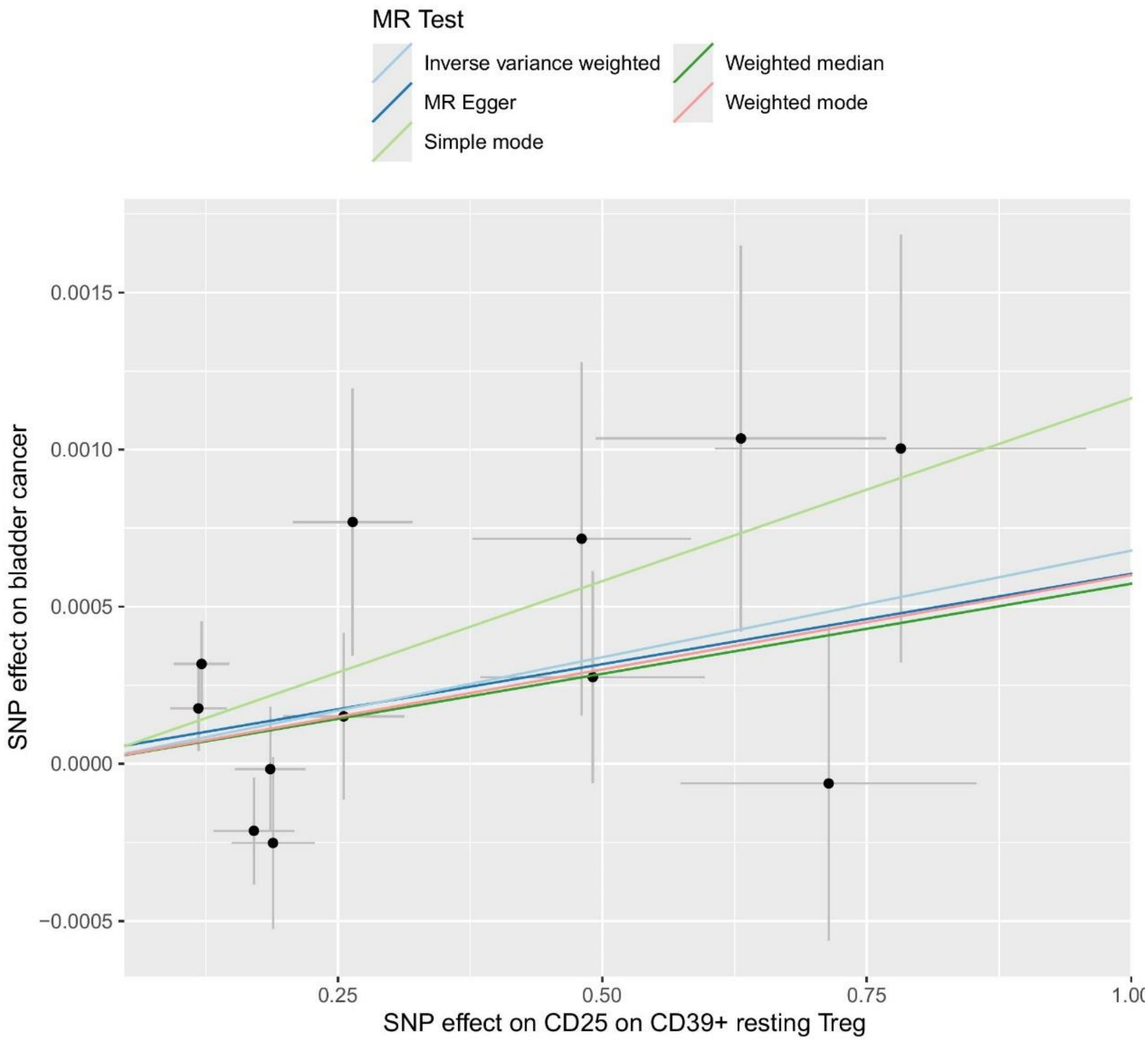

MR Test
Inverse variance weighted
MR Egger
Simple mode
Weighted median
Weighted mode
0.0015
0.0010
0.0005
0.0000
−0.0005
0.25
0.50
0.75
SNP effect on bladder cancer
SNP effect on CD25 on CD39+ resting Treg

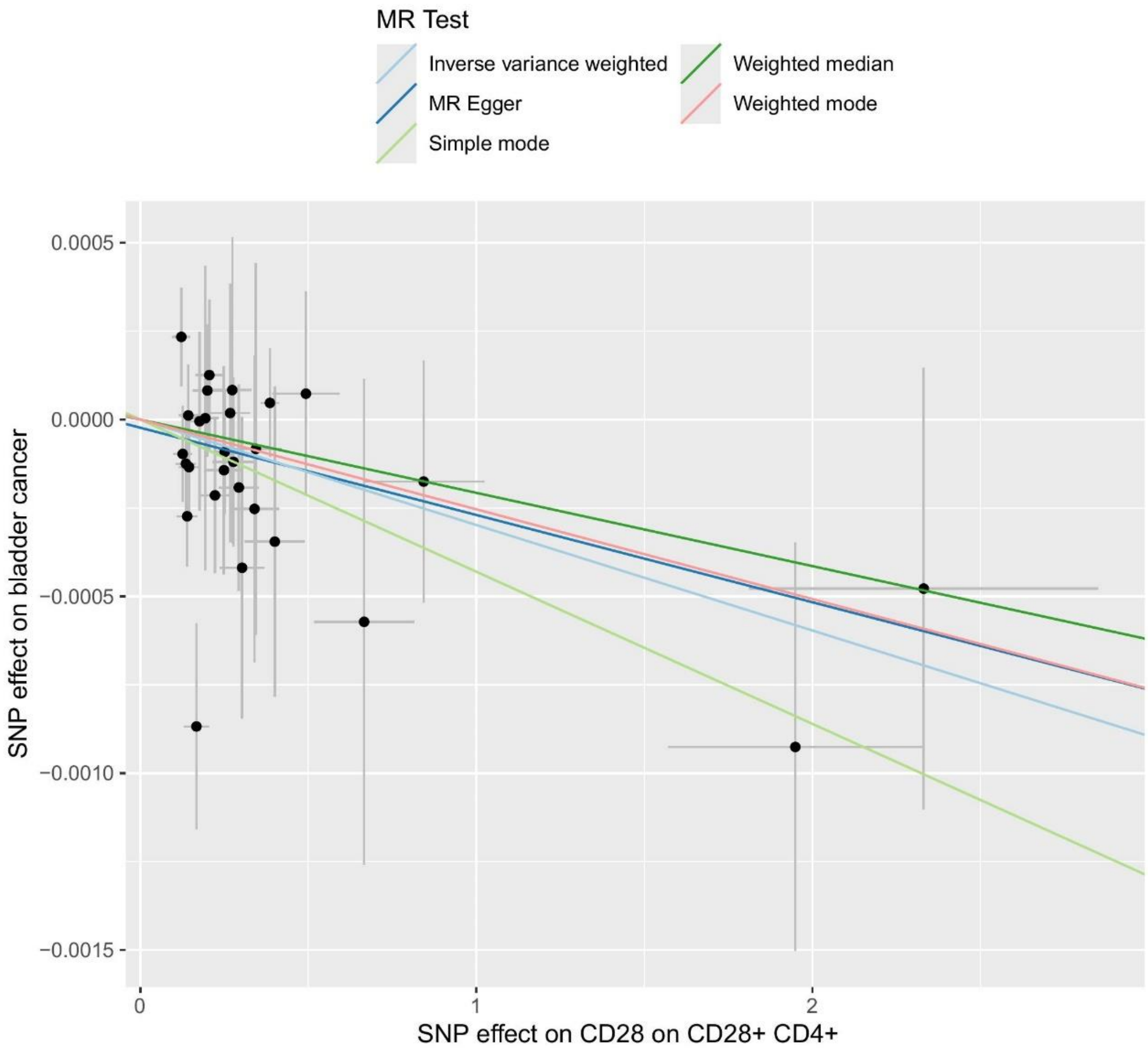
MR Test
Inverse variance weighted
MR Egger
Simple mode
Weighted median
Weighted mode
0.0005
0.0000
−0.0005
−0.0010
−0.0015
SNP effect on bladder cancer
0
1
2
SNP effect on CD28 on CD28+ CD4+

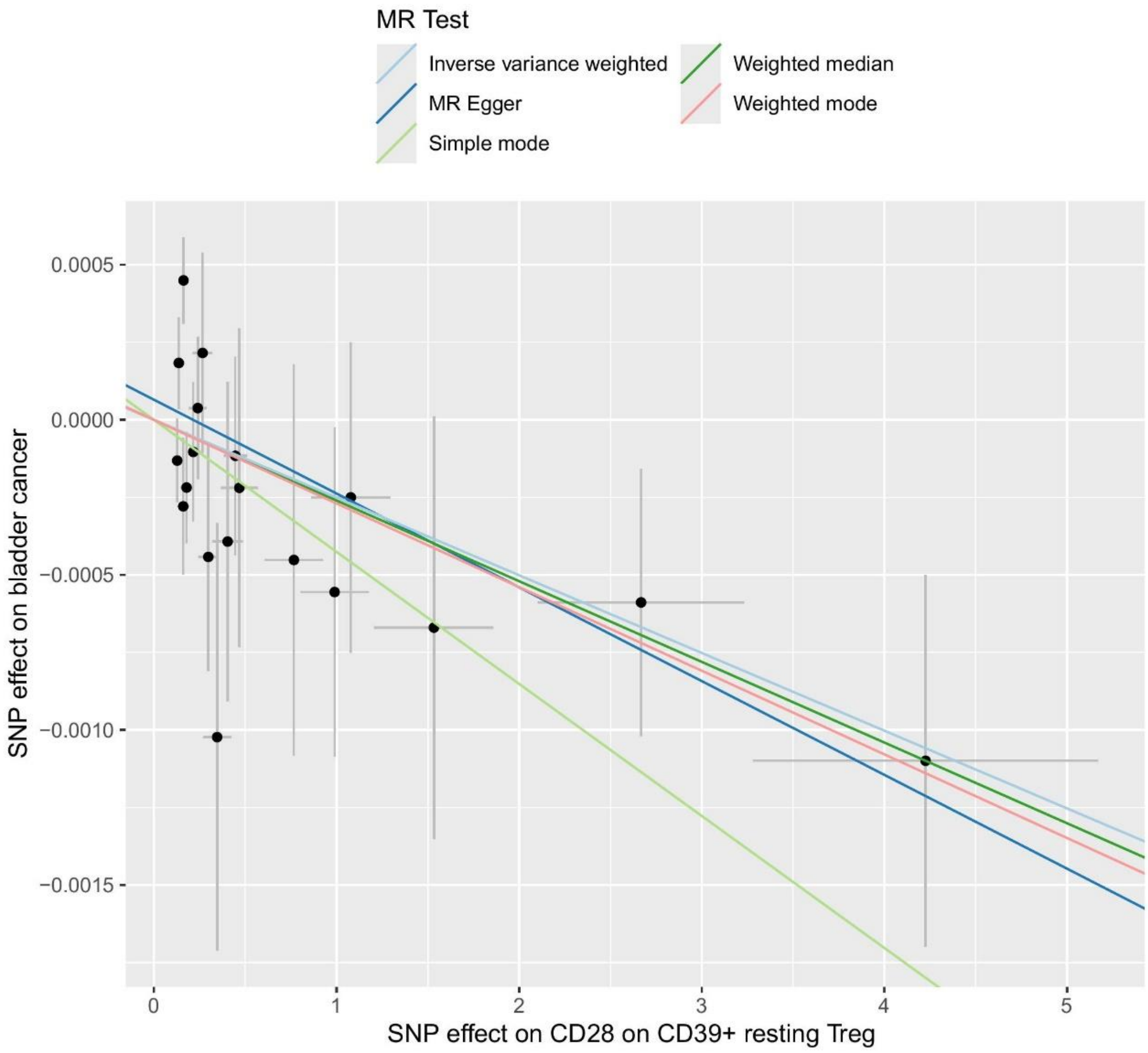
MR Test
Inverse variance weighted
MR Egger
Simple mode
Weighted median
Weighted mode
0.0005
0.0000
−0.0005
−0.0010
−0.0015
SNP effect on bladder cancer
0
1
2
3
4
5
SNP effect on CD28 on CD39+ resting Treg

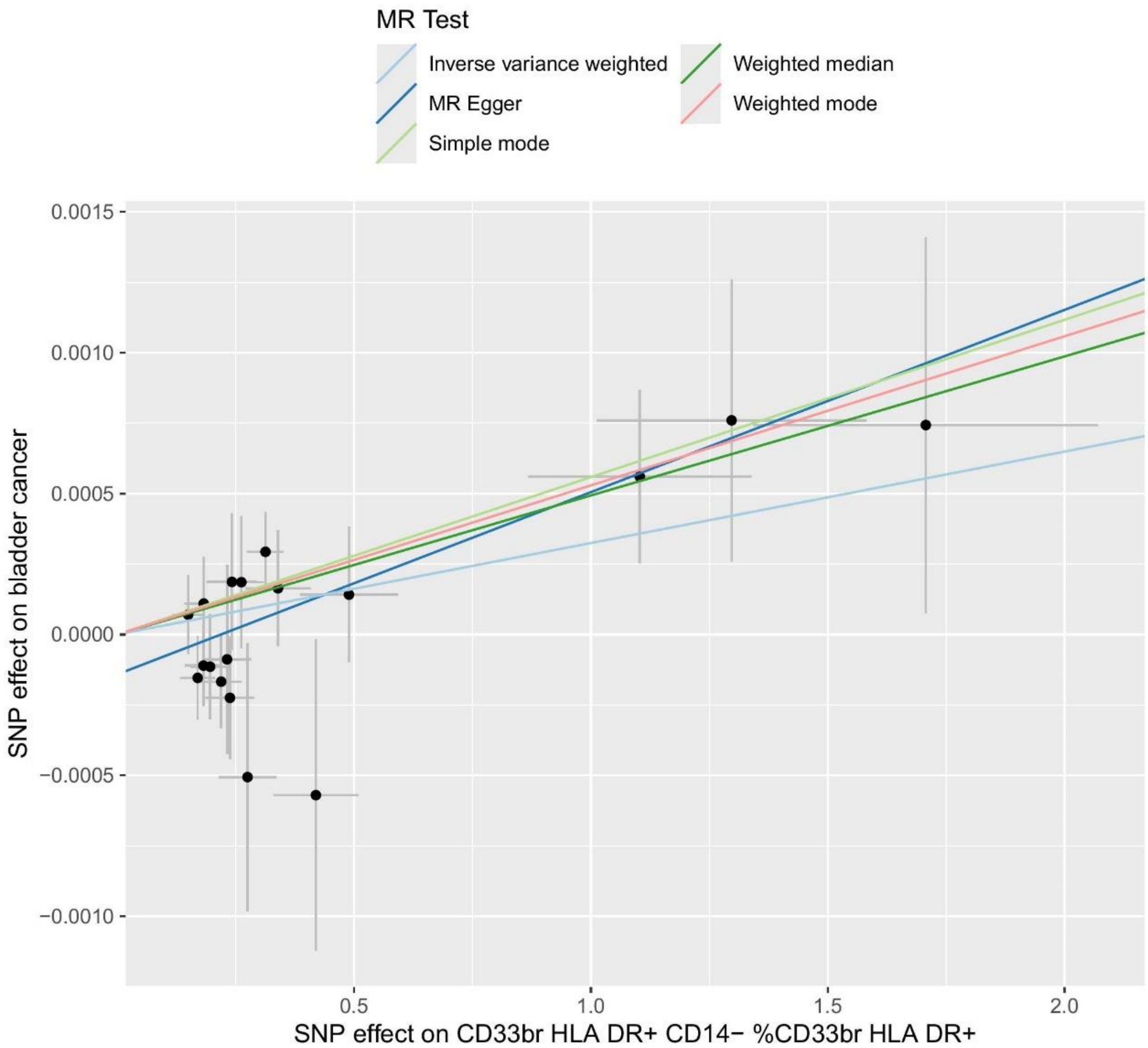

MR Test
Inverse variance weighted
MR Egger
Simple mode
Weighted median
Weighted mode
0.0015
0.0010
0.0005
0.0000
−0.0005
−0.0010
SNP effect on bladder cancer
0.5
1.0
1.5
2.0
SNP effect on CD33br HLA DR+ CD14− %CD33br HLA DR+

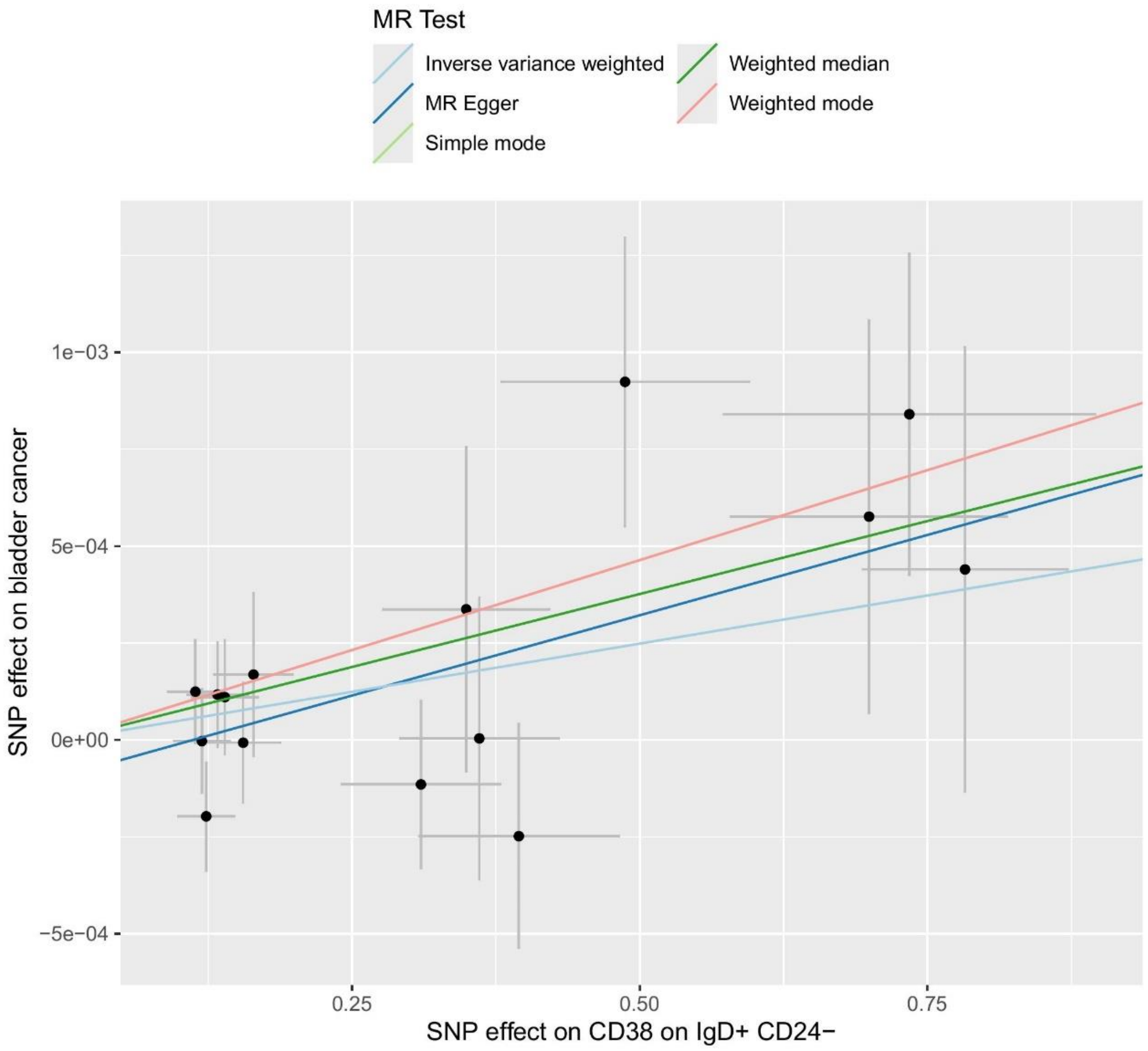
MR Test
Inverse variance weighted
MR Egger
Simple mode
Weighted median
Weighted mode
1e−03
5e−04
0e+00
−5e−04
0.25
0.50
0.75
SNP effect on bladder cancer
SNP effect on CD38 on IgD+ CD24−

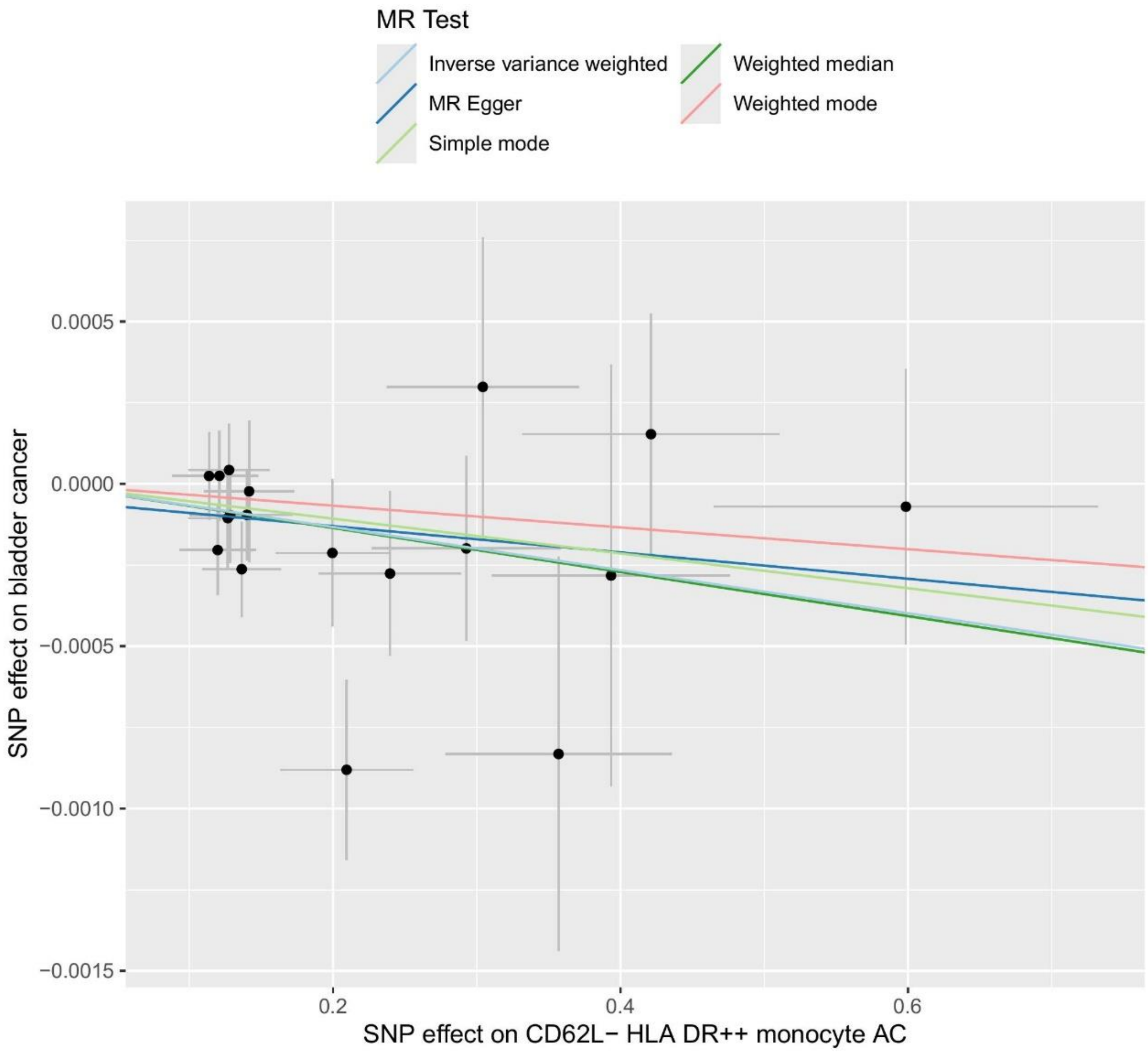

MR Test
Inverse variance weighted
MR Egger
Simple mode
Weighted median
Weighted mode
SNP effect on bladder cancer
0.0005
0.0000
−0.0005
−0.0010
−0.0015
0.2
0.4
0.6
SNP effect on CD62L− HLA DR++ monocyte AC

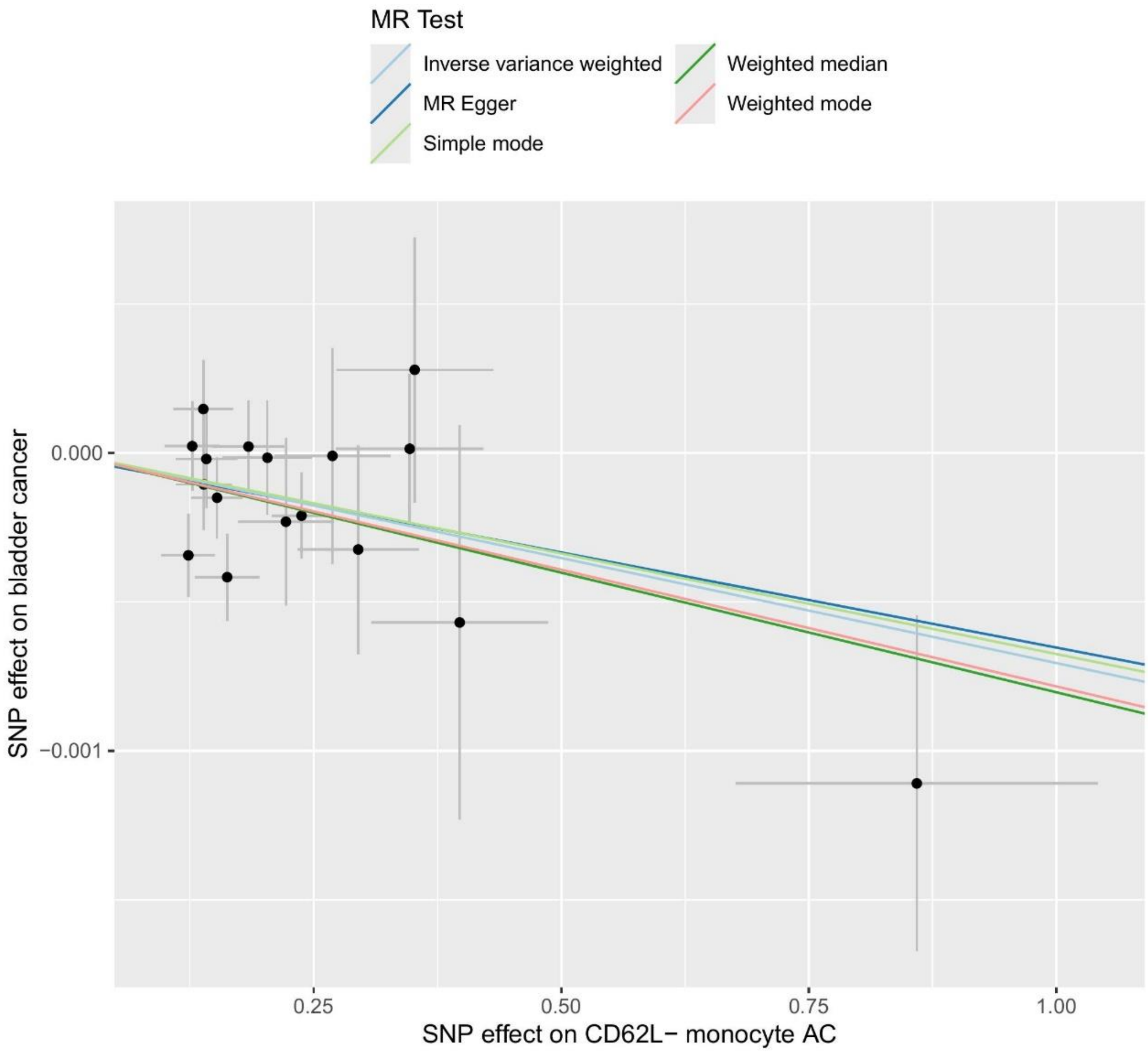
MR Test
Inverse variance weighted
MR Egger
Simple mode
Weighted median
Weighted mode
0.000
−0.001
SNP effect on bladder cancer
0.25
0.50
0.75
1.00
SNP effect on CD62L− monocyte AC

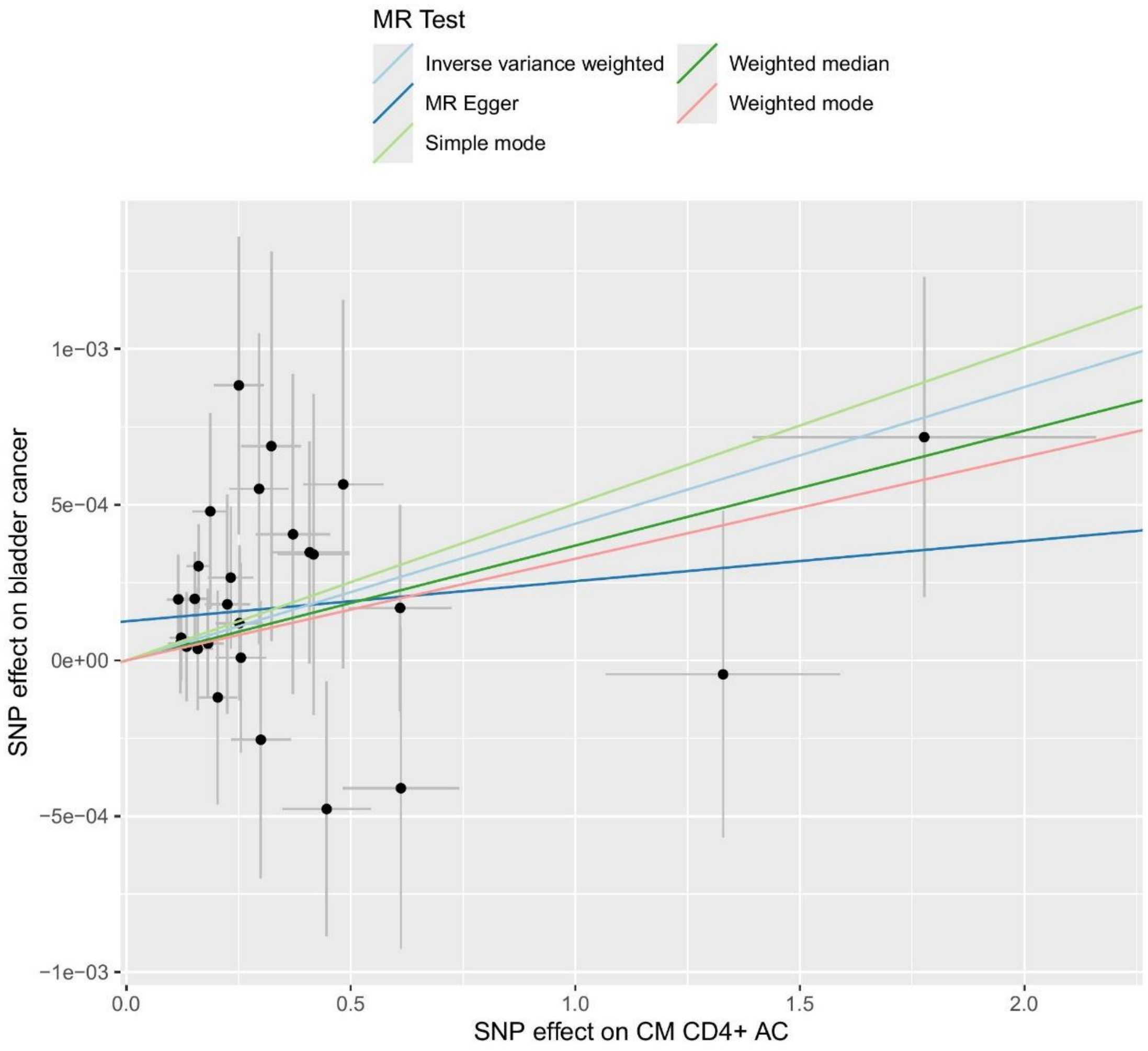
MR Test
Inverse variance weighted
MR Egger
Simple mode
Weighted median
Weighted mode
SNP effect on bladder cancer
1e−03
5e−04
0e+00
−5e−04
−1e−03
0.0
0.5
1.0
1.5
2.0
SNP effect on CM CD4+ AC

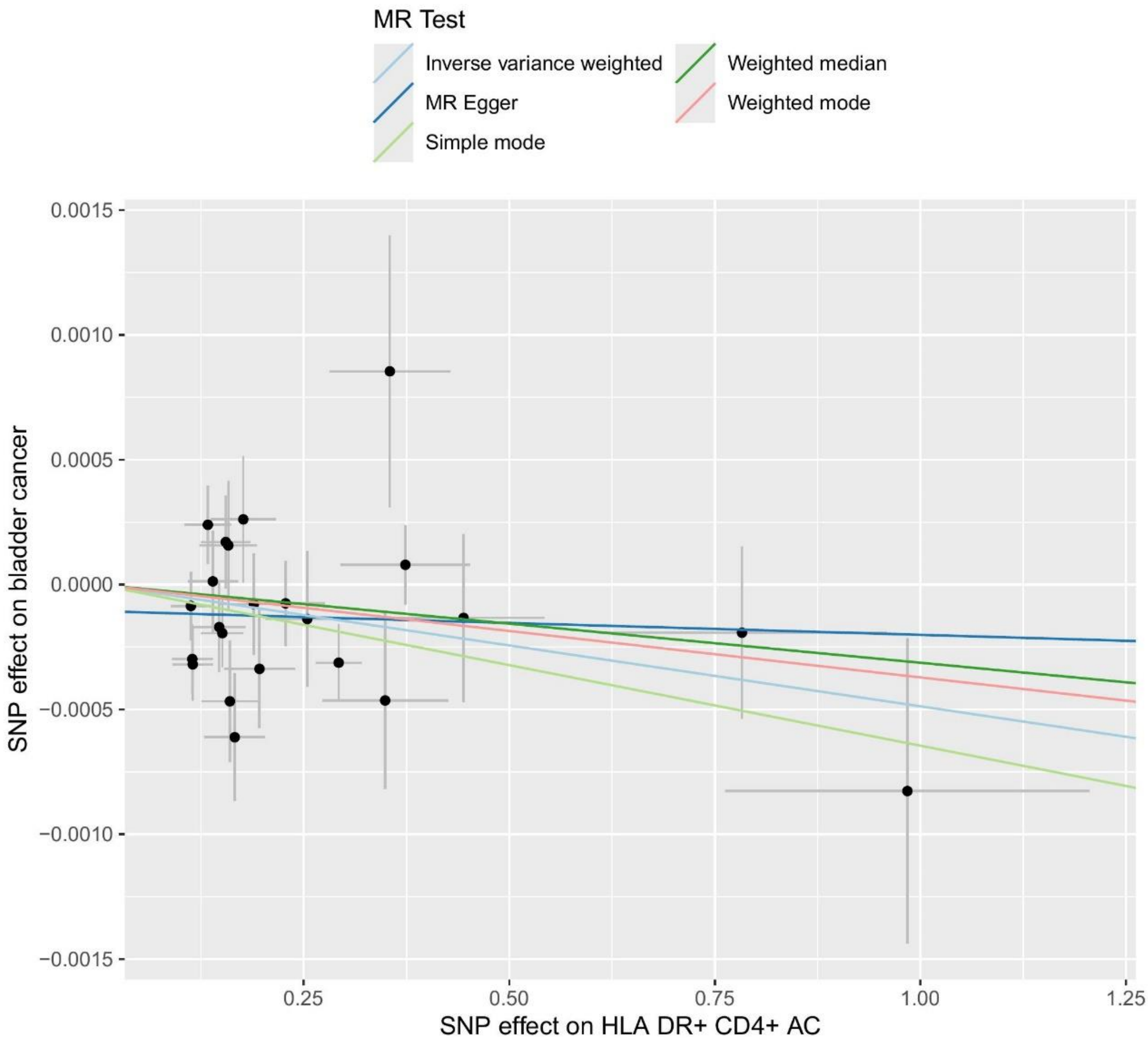

MR Test
Inverse variance weighted
MR Egger
Simple mode
Weighted median
Weighted mode
0.0015
0.0010
0.0005
0.0000
−0.0005
−0.0010
−0.0015
SNP effect on bladder cancer
0.25
0.50
0.75
1.00
1.25
SNP effect on HLA DR+ CD4+ AC

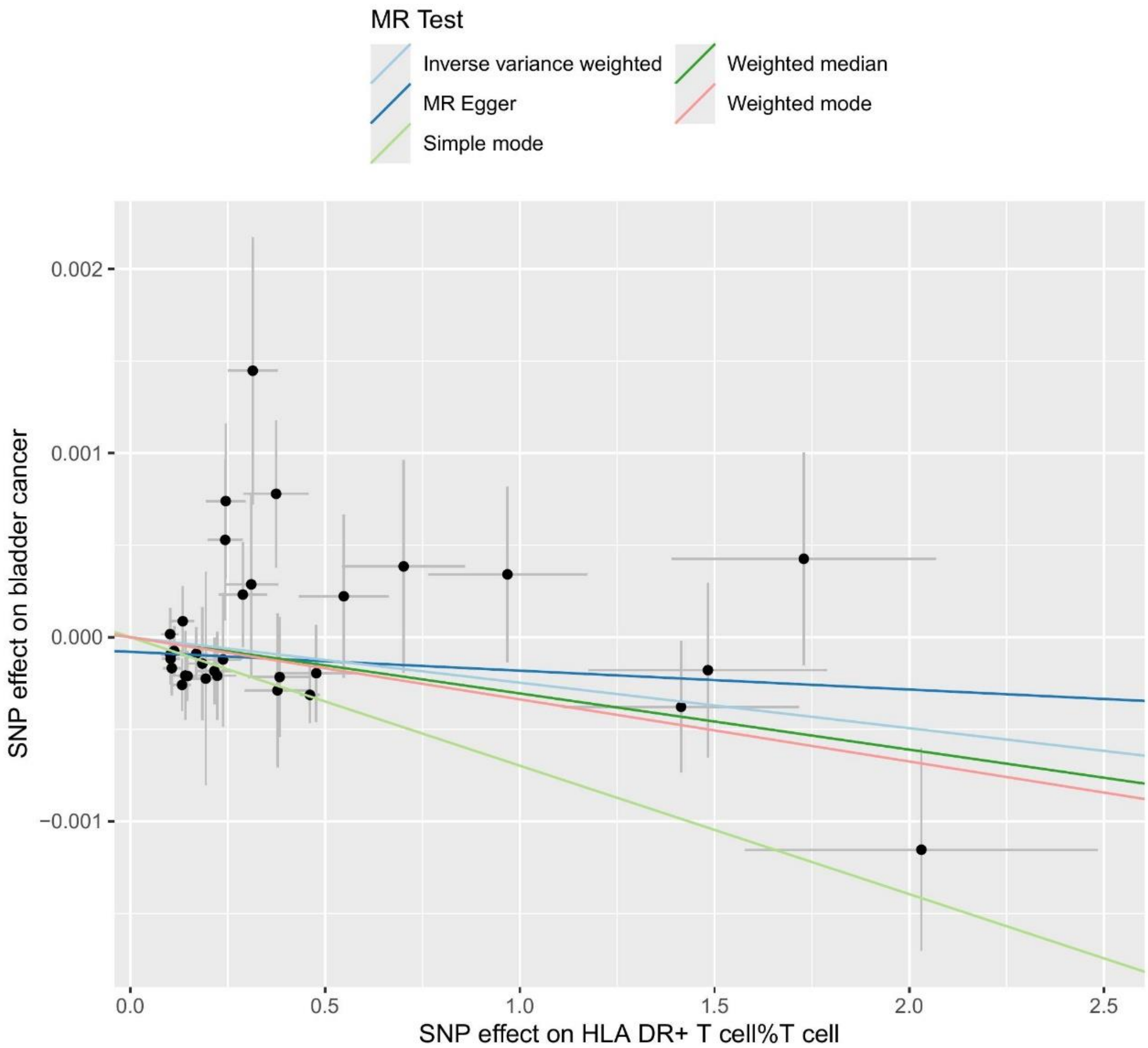
MR Test
Inverse variance weighted
MR Egger
Simple mode
Weighted median
Weighted mode
0.002
0.001
0.000
−0.001
SNP effect on bladder cancer
0.0
0.5
1.0
1.5
2.0
2.5
SNP effect on HLA DR+ T cell%T cell

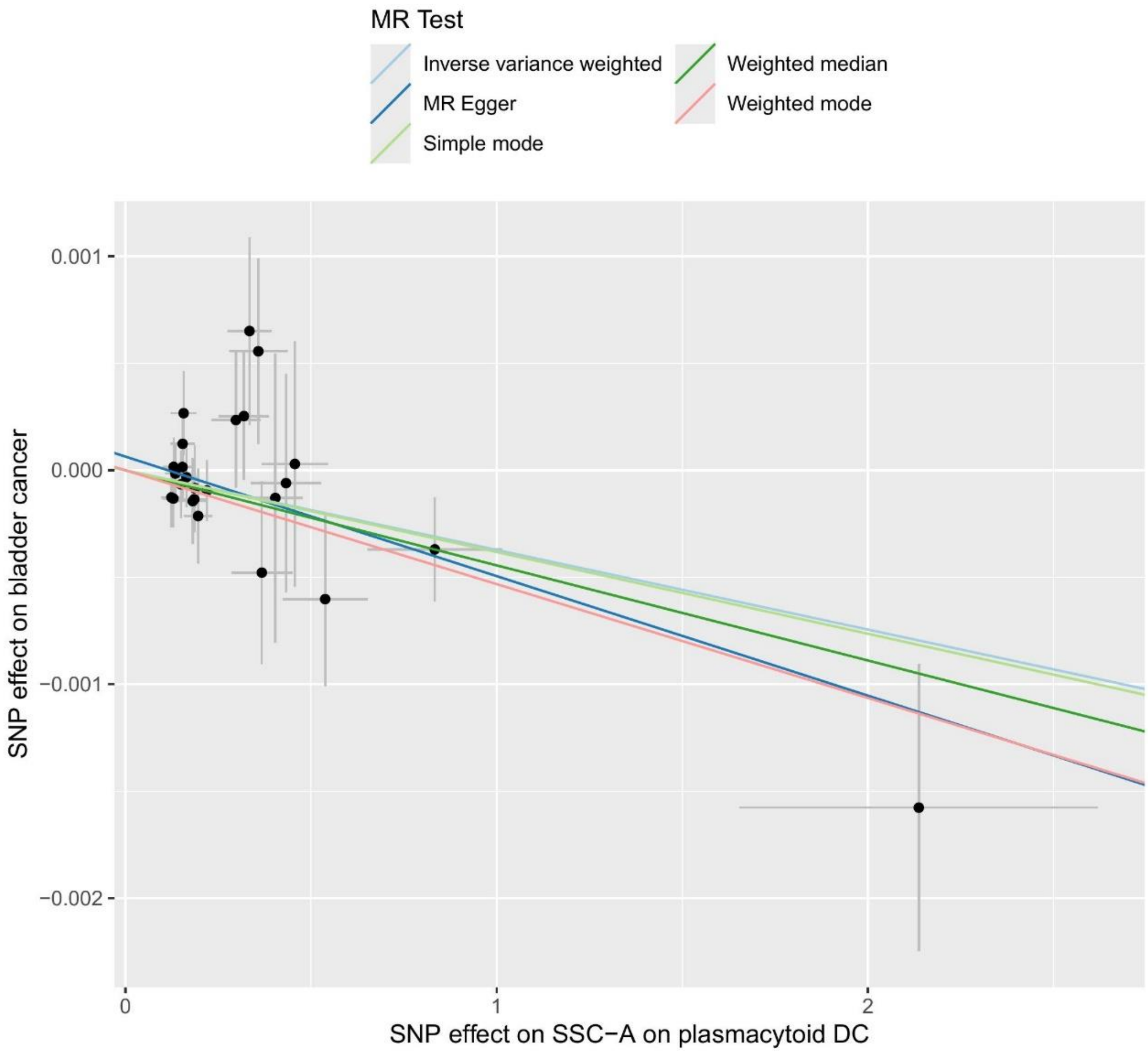

Supplementary Figure 2 Scatterplot of genetic association between 16 immune traits and bladder cancer.
